# Driving Effects of Large-Scale Sand Mining Activities on Bacterial Communities in Subtropical River Sediments—A Case Study of the Jialing River

**DOI:** 10.3390/microorganisms13091998

**Published:** 2025-08-27

**Authors:** Jia Xia, Tuo Zhang, Fei Xu, Maojin Huang, Fubin Zhang

**Affiliations:** 1 College of Environmental Science and Engineering, China West Normal University, Nanchong 637009, China; xiajia0212@163.com (J.X.); ilffvm99@163.com (F.X.); hmj666040604@163.com (M.H.); 2 School of Environmental and Life Sciences, Nanning Normal University, Nanning 530001, China; zhangtuo@nnnu.edu.cn

**Keywords:** Jialing River, large-scale sand mining, sediment, bacteria, community diversity, functional prediction

## Abstract

Sand mining activities can significantly impact the microecology of rivers. Scientific studies are needed for the effective protection and restoration of river ecosystems impacted by sand mining activities. In this study, we used high-throughput sequencing technology to analyse the structure and function of sediment bacterial communities in three river habitats of the Jialing River Basin, namely, a natural river channel (no sand mining activities), a channel with continuous large-scale sand mining activities, and a channel in which sand mining had been terminated one year prior, as well as to analyse the main constraints leading to changes in sediment bacterial communities. The results revealed that the dominant bacteria in the different sand mining environments of the Jialing River were Proteobacteria, Chloroflexi, and Acidobacteria, and that total organic carbon (TOC), moisture content (MC) and total nitrogen (TN) were the main limiting factors affecting the structure of the bacterial community. In addition, large-scale sand mining activities caused significant changes (*p* < 0.05) in major secondary functions, such as energy metabolism, cofactor and vitamin metabolism, and translation. In summary, the persistence of large-scale sand mining activities led to heterogeneous changes in sediment bacterial community structure and function, which had an important impact on the stability of the ecosystem in the Jialing River Basin.

## 1. Introduction

The amount of sand transported by rivers is an important indicator of soil erosion and conservation in the river basin, as well as of the improvement of river channels. With the accelerated process of global economic integration, the promotion of various types of construction activities has gradually increased the demand for sand. In most rivers, the impact of large-scale mining activities involves a significant reduction in sand delivery, deterioration of water quality, and negative impacts on riparian vegetation and other aspects [1,2,3]. Although the Chinese government fully recognizes the harmful effects of large-scale sand mining activities on the ecological damage of rivers, management authorities and researchers are paying increasing attention to this problem [4,5]. However, the expansion of the scale of sand mining activities has jeopardized the safety of flood control and navigation in the Yangtze River Basin and has resulted in serious river pollution and water ecological health problems [6]. In recent years, uncontrolled and indiscriminate mining activities have been brought under control, but the impacts that have been caused cannot be eliminated in the short term [7].

Water ecosystems play critical roles in maintaining water quality and nutrient recycling and providing energy for human survival and development, and large-scale sand mining activities can affect riverine water ecosystems to some extent [8,9,10]. At the same time, in the sand mining process, pollutants, such as oil pollution, are discharged into the natural water body, and with the flow of the water body constantly spreading, microorganisms in the water body are attached to the particles of pollutants and deposited by a variety of mechanisms, thereby affecting or changing the structure of the microbial community in the river sediment [11]. The diversity of sediment microbial communities in rivers with sand mining activities is lower than that in natural rivers, and more oil-degrading and toxicity-reducing microflora are found in sediment microbial communities affected by sand mining activities [12,13]. River sediments are fundamental components of the natural environment, transport elements, and are important pollutant reservoirs in water bodies and on land, with specific variability in response to disturbances in watersheds [14,15]. Therefore, the diversity of sediment microbial community structure and microbial community function in different environments can help explain the relationships between microbes and environmental impact factors [16,17,18].

The Jialing River is a tributary of the Yangtze River basin with the largest area, one of the key sand-producing rivers, and an important area for the sediment source of the Three Gorges Reservoir. Its runoff sediment comes from the Bailongjiang River, the Qiujiang River, the Fujiang River, and the area of the confluence of the three rivers [19,20]. Many studies have shown that with the development of sand mining activities, such as damming, there have been significant changes in various aspects of sand production patterns, flooding, and sand transport in watersheds, which have severely affected natural riverbeds, natural wetlands, and water quality [21,22,23]. Previous studies have shown that sand mining activities reduce the diversity of bacterial communities in river sediments. Moreover, sand mining activities lead to an increase in the abundance of Proteobacteria and a decrease in the abundance of Chloroflexi [24,25]. The abundance of Proteobacteria can indicate the extent of oil pollution, whereas the abundance of Chloroflexi can indicate the ability of bacterial communities to perform photosynthetic carbon sequestration [26]. However, few studies have compared the changes in bacterial community structure before sand mining activities, during sand mining activities, and one year after the cessation of sand mining activities. The composition and function of sediment microbial communities in watersheds disturbed by sand mining activities remain unclear. Therefore, the mechanisms by which sand mining activities affect sediment microbial communities require further investigation. In addition, studies have shown that the restoration of habitats or stream channels in watersheds where sand mining activities have taken place is lagging, and in severe cases, streams may lose their natural resilience [27]. Therefore, in this study, we examined three different types of river channels in the Jialing River basin: natural river channels, continuously dredged river channels, and river channels that had ceased dredging for one year. We employed high-throughput sequencing technology and bioinformatics analysis methods to study the evolutionary trends of bacterial community characteristics in sediments under different sand mining conditions in the Jialing River basin. This was done to reveal the extent to which large-scale sand mining activities impact the structure and function of sediment bacterial communities and to explore the effects of such activities on the microbial communities in sediments. We aim to provide a scientific basis for ecological environmental protection and sand mining management in the Jialing River Basin by controlling the mining intensity threshold, mandatory management of the restoration period, and microbial-driven river restoration techniques.

## 2. Materials and Methods

**Overview of the study area.** The study area is located in the middle and lower reaches of the Jialing River in the upper reaches of the Yangtze River (105°55′32″~106°5′21″ E, 31°38′3″~30°44′17″ N). The Jialing River originates from the southern foot of the Qinling Mountains, and its flow passes through four provinces and cities, namely, Shanxi, Gansu, Sichuan, and Chongqing, and eventually joins the Yangtze River system. With a drainage area of 159,800 km^2^ and a total length of approximately 1119 km, the Jialing River is the largest tributary in the Yangtze River system. The region has a subtropical monsoon climate, with an average annual precipitation of 931 mm and an average temperature of approximately 17.1 °C throughout the year. Its sediment mainly originates from the confluence of the main stream, the Qiujiang River, and the Fuljiang River, which is the basin with the greatest amount of sand transport in the upper reaches of the Yangtze River. The Jialing River Basin is a key sand-producing area, and large-scale sand mining activities in the river have affected and damaged the stability of the river and the ecological environment within the basin [28].

**Figure 1:**
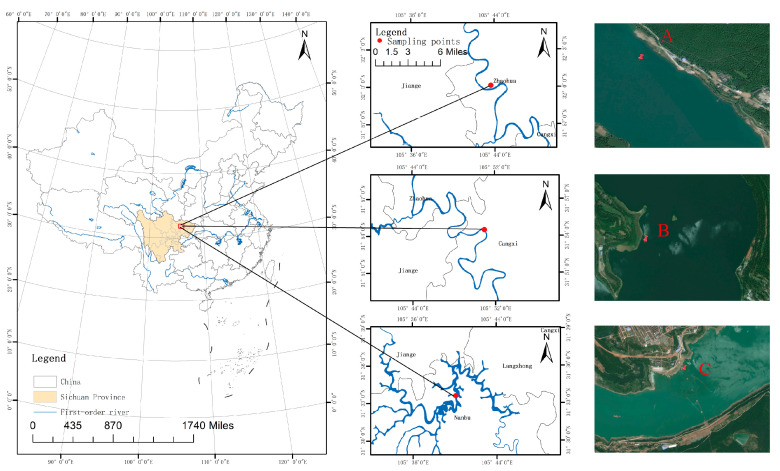
Study area and sampling site information. A represents a natural river channel, B represents a river channel with ongoing large-scale sand mining activities, and C represents a river channel in which sand mining activities were terminated one year prior.

**Sampling point layout and sample collection.** One sampling point was set in each of the three river sections of the Jialing River, namely, Yuanxi town, Xiaxin town, and Hutiao town, and the specific information of the sampling points is shown in Figure 1. The three sampling points represent three typical different sand mining environments: Yuanxi town is a natural river channel (no sand mining activities), Xiaxin town is a large-scale sand mining river channel with continuous large-scale sand mining, and Hutiao town is a channel where the sand mining had been terminated one year prior; these towns are denoted as A, B, and C, respectively.

Sample collection was carried out in June 2021, and one sediment sample was collected from each point using a cleaned and sterilized sediment dwelling. Each sample was collected at a depth of approximately 10 cm, according to a gradient of every 2 cm, i.e., 5 samples for one sampling point, and a total of 15 samples for 3 sampling points, which were labelled from shallow to deep as A1~A5, B1~B5, and C1~C5, respectively, and brought back to the laboratory under refrigeration to be stored in an ultralow-temperature refrigerator at −80 °C (Suzhou, China) and then used for the determination of physical and chemical indices of the sediments and the analysis of the diversity of bacterial communities in the sediments.

**Determination of physical and chemical indices of the sediments.** The S content was determined by flame atomic absorption photometry (SC; Instrument Model: atomic absorption spectrophotometer Z-2300, Hitachi, Ltd., Tokyo, Japan) [29]. The pH was determined by the electrode potential method (HJ 613-2011) (Instrument Model; pHB-4, Shanghai Yidian Scientific Instrument Co., Ltd, Shanghai, China). The sediment moisture content (MC; Instrument Model: Electrothermal blast drying oven WGL-45B, Tianjin Test Instrument Co., Ltd., Tianjin, China) [30] was determined using the air-dried soil sample drying method by drying naturally air-dried soil samples at 105 °C until a constant weight was reached. The total organic carbon (TOC; Instrument Model: COD Eliminator RD 125, Tintometer-Lovibond, Dortmund, Germany) content in the sediment was determined by the cauterization method [31]. The total phosphorus (TP) and total nitrogen (TN) contents were determined by UV spectrophotometry (HJ 636-2012; Instrument Model: Ultraviolet and Visible Spectrophotometer TU-1901, Beijing Puxi General Instrument Co., Ltd, Beijing, China) [32].

**Determination of sediment microorganisms.** Sample DNA was first extracted using the Mag-Bind^®^ Soil DNA Kit (Omega Biotek, Norcross, GA, USA), after which the DNA concentration and purity were tested (Measure the UV absorption values at 260 nm and 280 nm. When the OD260/OD280 ratio is between 1.8 and 2.0, the DNA purity meets the requirements, and DNA integrity was examined using 1% agar-gel electrophoresis. The DNA was fragmented with a Covaris M220 (Genetics, Beijing, China) to screen a fragment of approximately 400 bp, and a paired-end library was subsequently constructed via NEXTFLEX Rapid DNA-Seq (Bioo Scientific, Austin, TX, USA). The sequences of the template DNA fragments were obtained by bridge PCR amplification on the basis of the library molecules and macrogenome sequencing (Majorbio Bio-Pharm Technology Co., Ltd., Shanghai, China) using the Illumina NovaSeq 1.0 (Illumina, San Diego, CA, USA) sequencing platform.

The sequences were quality clipped using fastp (version 0.20.0) to retain high-quality pair-end reads and single-end reads13 [33]. The optimized sequences were spliced and assembled using the software MEGAHIT (version 1.1.2), and ORFs were predicted for contigs larger than 300 bp using MetaGene as the final assembly result [34,35]. The genes with nucleic acid lengths greater than or equal to 100 bp were then selected and translated into amino acid sequences, and the predicted gene sequences of all the samples were clustered using CD-HIT (version 4.6.1) to construct a nonredundant gene set (parameters: 90% identity, 90% coverage). The final analysis was performed on the BioCloud platform by Meji Corporation (Itasca, IL, USA), which involved comparing nonredundant gene sets with relevant databases [36].

**Bioinformatics Statistics and Analysis.** Experimental sequencing and data analysis were performed using the BioCloud platform by Meggie. The data was classified into OTUs, determined by 100% sequence similarity using R packages, and the Venn diagram was drawn. Species annotations were obtained using Diamond (version 0.8.35) by comparing the amino acid sequences of the nonredundant (NR) gene set with the taxonomic information database corresponding to the NR database, and then the abundance of the species was calculated using the sum of the corresponding gene abundances for the species. Bacterial community alpha diversity indices (including the abundance-based coverage estimator (ACE), Shannon, and Simpson indices) were analysed with mothur-1.30 software. The relative abundance of primary and secondary functions of the bacterial community was analysed with PICRUSt2 software (version 2.2.0), annotated against the NR and KEGG databases (the database itself has several limitations: the analysis results can be used as a reference basis, if necessary, and can be combined with macrogenome sequencing and other analytical tools for further research), and Python 2.0 for LEfSe (LDA Effect Size) analysis. Redundancy analysis (RDA) was performed, and the results were plotted using RDA in the R language vegan package.

## 3. Results

### 3.1. Physical and Chemical Properties of the Sediments at Each Sampling Point

**Figure 2:**
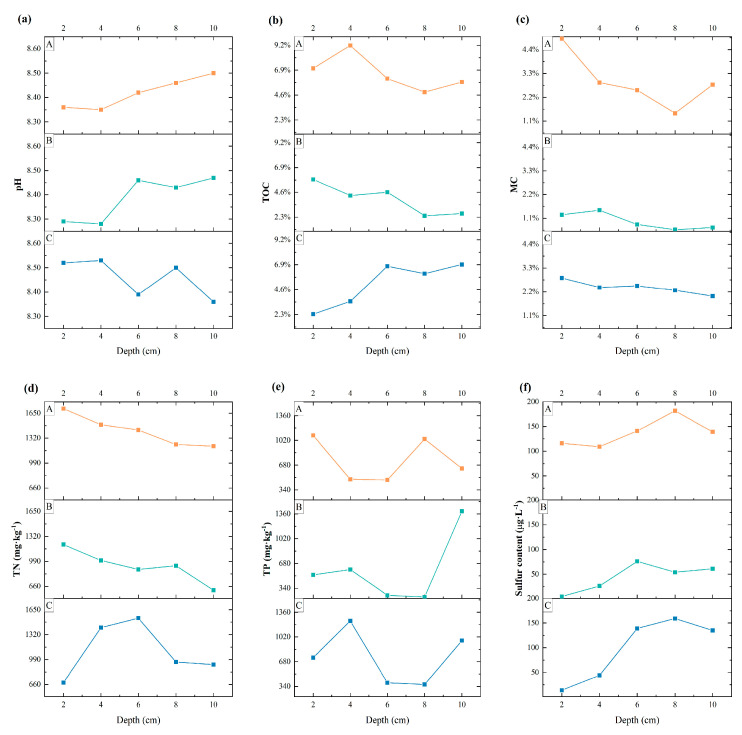
Comparison of the physical and chemical properties of sediment samples at three sites (A, B, and C). (**a**): pH. (**b**): total organic carbon, TOC. (**c**): moisture content, MC. (**d**): total nitrogen, TN. (**e**): total phosphorus, TP. (**f**): S content, SC. A represents a natural river channel, B represents a river channel with ongoing large-scale sand mining activities, and C represents a river channel in which sand mining activities were terminated one year prior. The horizontal coordinate in the figure represents the depth of the sediment, and the scale of the interface in contact with water is 0.

As shown in Figure 2, the physical and chemical properties of the sediment varied significantly among the different sand mining environments and different sampling depths. The pH values of the sediment at the three sampling sites were weakly alkaline, and the TN content was generally high, ranging from 927 to 1417 mg·kg^−1^. For the six types of physical and chemical indicators, namely, pH, TOC, MC, TN, TP, and SC, at different sampling points, the level or content of each indicator at sampling point B tended to be lower than that at sampling point A, with the lowest values of the five physical and chemical indicators, namely, pH, MC, TN, TP, and S, all at sampling point B. Except for the pH indicator, the other indicators presented the phenomenon of A > C > B. In terms of the sediments at different depths at sampling site C, the TN content of the sediments at sampling sites A and B tended to decrease with increasing depth, whereas the TN content of the sediments at sampling site C tended to increase and then decrease with increasing depth. The TOC content of the sediments at sampling sites A and B tended to decrease with increasing depth, whereas that of the sediment at sampling site C tended to increase. The MC of all three sites tended to decrease with increasing depth. The magnitude of the SC indicator values as a whole tended to increase with increasing depth.

### 3.2. Bacterial Community Structure and Differences

#### 3.2.1. Bacterial OTU Number

**Figure 3:**
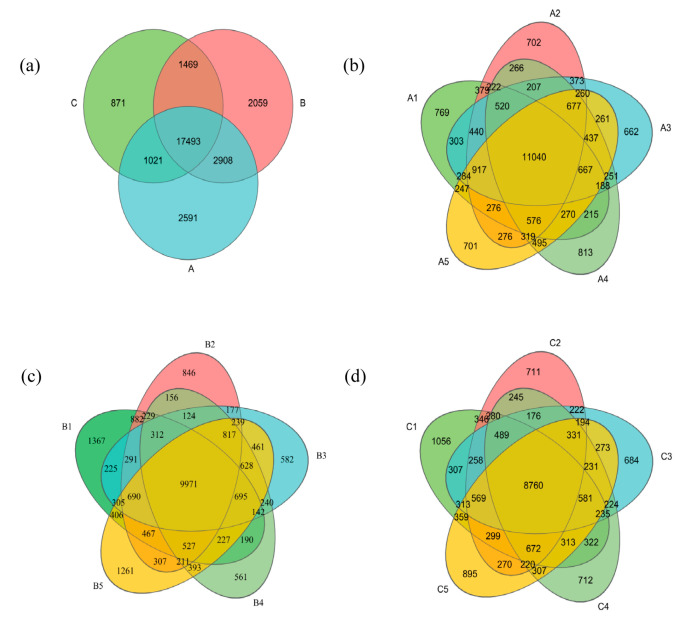
Comparison of the OTUs of bacteria in sediments at different sampling points. Number of sediment bacterial OTUs at each site, specific to a site, or shared by two or five sites (the number of OTUs within each site was calculated after the sequences obtained from the three replicates from each site were merged). The median value of the three values obtained in the triplicate analysis is indicated as the value of the replicate. A represents sampling sites in a natural river, B represents sampling sites in a channel with continuous sand mining, and C represents sampling sites in a channel where the sand mining has been terminated one year prior; (**a**) comparative analyses of sampling sites A, B, and C; (**b**) comparisons between sampling sites at different depths at sampling site A; (**c**) comparisons between sampling sites at different depths at sampling site B; and (**d**) comparisons between sampling sites at different depths at sampling site C.

The Venn diagram visualizes the number of OTUs unique to and shared by a total of 15 samples from three different types of sand mining environments. As shown in Figure 3, the three sampling points were clustered into OTUs after optimizing the number of sequences by high-throughput sequencing, and a total of 67,986 OTUs were identified. Of these OTUs, the three sampling points had a total of 17,493 OTUs, and with unique OTU counts of 2591 at Site A (Percentage: 9.12%), 2059 at Site B (Percentage: 7.25%), and 871 at Site C (Percentage: 3.07%). Figure 3c,d shows that the two sites were affected by large-scale sand mining activities. Compared with those in B1, the numbers of unique OTUs in B2, B3, and B4 were significantly lower by 38.11%, 57.43%, and 58.96%, respectively. Compared with those in C1, the numbers of OTUs in C2, C3, and C4 were also significantly lower by 32.67%, 35.23%, and 32.58%, respectively, and tended to decrease first and then increase with increasing depth. That is, in the vertical direction, there was also a large disturbance to the sediment bacterial community by large-scale sand mining activities, with the degree of disturbance decreasing with depth. The above results indicate that large-scale sand mining activities have a persistent and significant effect on the distribution of the number of bacterial OTUs in the sediments of the Jialing River Basin.

#### 3.2.2. Alpha Diversity of the Bacterial Community

A comparison of alpha diversity indices was conducted on bacterial communities in sediment samples from three different sand mining environments, and the results are shown in Table 1. The Ace index indicated that the richness of bacterial communities in sediments at the same depth decreased in the following order: natural river A, sand mining river B, and river C, where sand mining had ceased for one year. Among these factors, large-scale sand mining had the least impact on the richness of bacterial communities in surface sediments. The richness of bacterial communities at sampling site B1 in the sand mining channel was 3.80% lower than that at natural channel A1, whereas the richness of bacterial communities at sampling site C1 in the channel one year after sand mining cessation was 11.42% lower than that at A1. Large-scale sand mining activities had the greatest impact on the richness of bacterial communities in the middle layer. The richness of the bacterial communities at sampling site B3 in the sand mining channel was 3.80% lower than that at sampling site A3 in the natural channel, and the richness of the bacterial communities at sampling site C3 in the channel one year after sand mining cessation was 11.42% lower than that at sampling site A3. A comprehensive analysis of the diversity indices (Shannon index and Simpson index) revealed that large-scale sand mining activities can significantly impact bacterial community diversity, leading to a reduction in diversity compared with that in natural river sections. However, one year after sand mining ceased, the diversity of bacterial communities in river sediments began to recover. Additionally, diversity indices indicate that large-scale sand mining activities have the greatest impact on the diversity of bacterial communities in mid-layer sediments. For example, according to the Simpson index, the diversity of bacterial communities in river section B3 was 31.67% lower than that in natural river section A3, and the diversity in B4 was 36.38% lower than that in A4.

In summary, large-scale sand mining activities can reduce the richness and diversity of bacterial communities in the sediment of the Jialing River to some extent, with a greater impact on mid-layer sediments than on surface and bottom sediments.

#### 3.2.3. Analysis of Bacterial Community Species Composition and Community Structure

**Figure 4:**
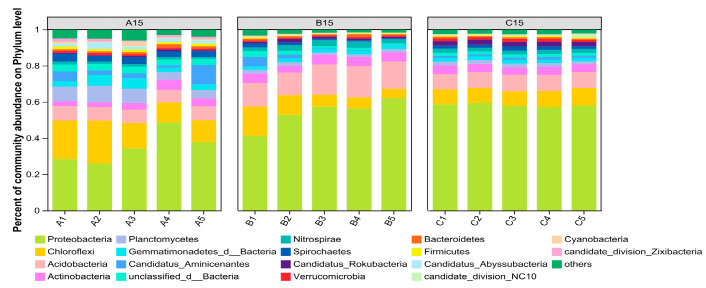
Histogram of the bacterial community structure in the sediments of the different interference treatment groups at the phylum level (the bacterial community structure of the top 10 most abundant phyla in the sediments). A represents a natural river channel; B represents a river channel with ongoing large-scale sand mining activities; and C represents a river channel in which sand mining activities were terminated one year prior.

Figure 4 shows the composition of the top 10 bacterial phyla in terms of relative abundance at the phylum level for the sediments from the three sand mining environments (The relevant data can be found in Supplementary Materials Tables S1–S3). As shown in Figure 4, the main dominant phylum in the three different sand mining environments was Proteobacteria (relative abundance, 45.08%), followed by Chloroflexi (relative abundance, 10.11%) and Acidobacteria (relative abundance, 9.51%). Comparative analysis of the main dominant bacterial phyla in different sand mining environments at the same depth revealed that the relative abundances of Proteobacteria and Acidobacteria in sites B and C, where sand mining was conducted, increased significantly compared with those in the natural river channel, whereas the relative abundance of Chloroflexi decreased compared with that in the natural river channel. An analysis of the relative abundance of dominant species in the sediments at different depths of sand mining revealed that the effects of sand mining on the bacterial communities at different depths of the sediments were in descending order: middle layer, deep layer, and surface layer. In addition, the relative abundance of Proteobacteria at sampling point C, where sand mining has been terminated for 1 year, is still high, whereas the relative abundance of Chloroflexi is still low. The results show that large-scale sand mining activities have a significant effect on the bacterial community structure in the vertical direction and that it is difficult to recover in a short period.

**Figure 5:**
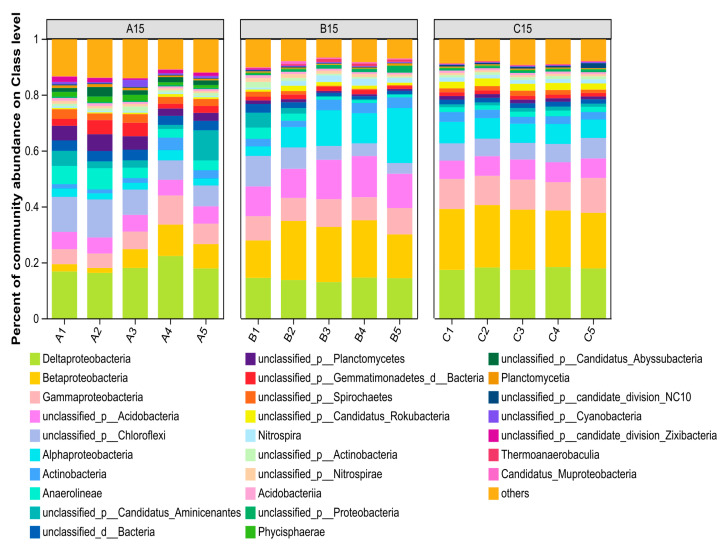
Histogram of the bacterial community structure in the sediments of the different interference treatment groups at the class level (the bacterial community structure of the top 10 most abundant taxa in the sediments at the class level). A represents a natural river channel; B represents a river channel with ongoing large-scale sand mining activities; and C represents a river channel in which sand mining activities were terminated one year prior.

Figure 5 shows the structure of the top 10 bacterial classes in terms of relative abundance at the compendium level for the sediments (The relevant data can be found in Supplementary Materials Tables S1–S3). As shown in Figure 5, the dominant species were Deltaproteobacteria (28.27%), Betaproteobacteria (26.45%), Gammaproteobacteria (15.31%), undefined Acidobacteria (14.58%), unclassified Chloroflexi_unclassified (12.76%), and Alphaproteobacteria (11.97%), and the relative abundances of the remaining classes were less than 5%. The relative abundance of bacteria in the surface sediment under different sand mining environments revealed that Betaproteobacteria, Gammaproteobacteria, Acidobacteria_unclassified, and Alphaproteobacteria were enriched in comparison with those in the natural channel and did not recover even 1 year after sand mining activities had been terminated. The relative abundance of Acidobacteria_unclassified in the middle layer increased significantly, which is consistent with the above analysis. This finding is consistent with the above analysis, which further indicates that the surface layer of sediments affected by sand mining activities is severely polluted by oil, which has an impact on the bacterial community structure in the vertical direction and is prone to inducing the dominant species in the middle layer to undergo heterogeneous changes.

**Figure 6:**
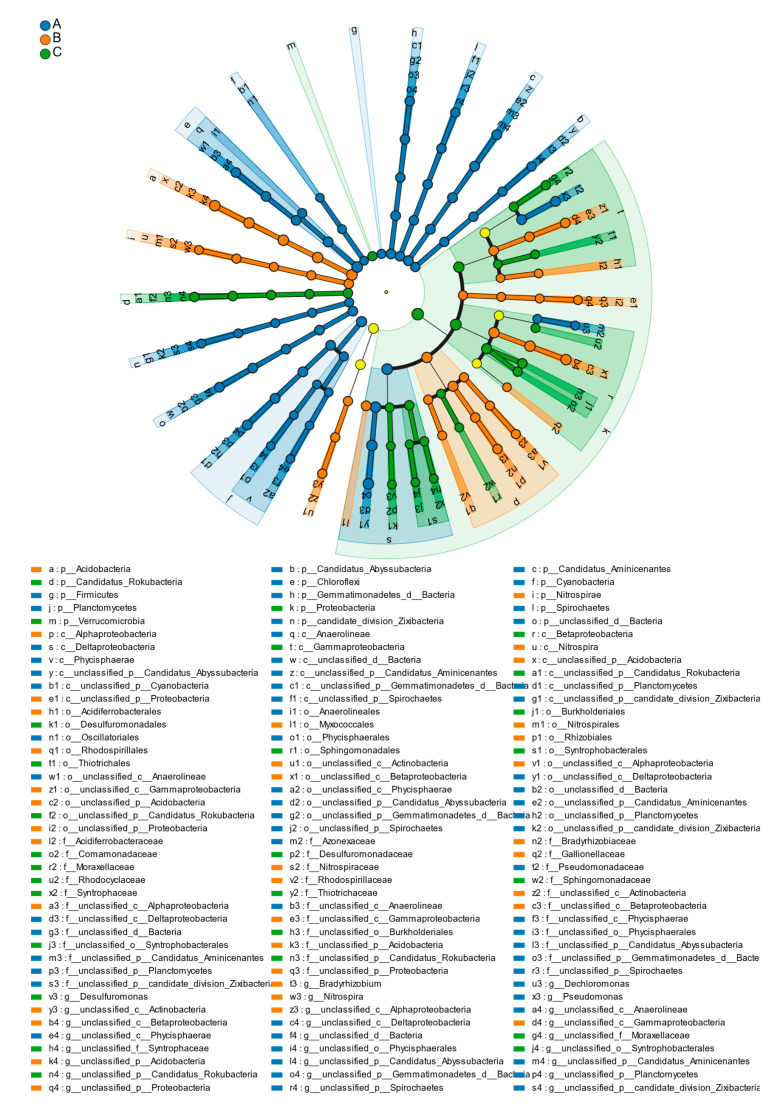
Analysis of the soil bacterial community in the different sediment types (A, Sampling sites in a natural river; B, Sampling points in a channel with continuous sand mining; C, Sampling points in a channel in which sand mining had been terminated 1 year prior, LAD score > 3.5). Odes in different colours indicate microbial groups that were significantly enriched in the corresponding group and had a significant effect on the differences between groups. Light yellow nodes indicate microbial groups that were not significantly different in different subgroups or that did not have a significant effect on the differences between groups. The letters “p, c, o, f, g” in the icon represent “Phylum, class, order, family, and genus”, respectively.

LEfSe (LDA effect size) can be used to compare multiple groups of samples to identify species with significant differences in abundance between groups (The relevant data can be found in Supplementary Materials Tables S1–S3). To determine the significant differences between sedimentary bacterial species in the three different sand mining environments, the tool was used to select the level of LDA score greater than 3.5 to draw phyla, class, order, family, and genus taxonomic bar charts, as shown in Figure 6. The results revealed that different dominant species were present at all types of sampling sites in the different sand mining environments, suggesting that the three different sedimentary environments varied in terms of degree of influence from large-scale sand mining activities and that the adaptive survival of each bacterial flora changed accordingly.

At the phylum classification level, at sampling site A, Candidatus_Aminicenantes, Chloroflexi, Firmicutes, Cyanobacteria, Gemmatimonadetes, Planctomycetes, Spirochaetes, Acidobacteria and Nitrospirae were found at sampling site B, and Proteobacteria, Verrucomicrobia and Rokubacteria were found at sampling site C. These bacteria are significantly dominant in each sand mining environment.

At the class level, Phycisphaerae, Anaerolineae, and Deltaproteobacteria were found at sampling site A; Alphaproteobacteria and Nitrospira were found at sampling site B; and Betaproteobacteria and Gammaproteobacteria were the dominant bacteria at sampling site C.

At the order level, Oscillatoriales, Anaerolineales, and Phycisphaerales were found at sampling site A; Acidiferrobacterales, Myxococcales, Rhizobiales, Nitrospirales, and Rhodospirillales were found at sampling site B; and Desulfuromonadales, Buekholderiales, Sphingomonadales, Desulfuromonadales, and Syntrophobacterales were found at sampling site C. These are notable potential fungi in each sand mining environment.

At the family level, Pseudomonadaceae and Azonexaceae at sampling site A and Acidiferrobacteraceae, Bradyrhizobiaceae, Rhodospirillaceae, Gallionellaceae, and Nitrospiraceae were found at sampling site B. Desulfuromonadaceae, Moraxellaceae, Comamonadaceae, Rhodocyclaceae, and Sphingomonadaceae were found at sampling site C. Thiotrichaceae is a prominent dominant bacterial family in various sand mining environments but was not found in this study.

At the genus level, *Pseudomonas* and *Dechloromonas* were found at sampling site A, and *Bradyrhizobium* and *Nitrospira* were found at sampling site B. At sampling site C, *Desulfuromonas* was a significantly dominant bacterium in the sand mining environment.

### 3.3. Correlations Between Bacterial Community Structure and Environmental Factors

**Figure 7:**
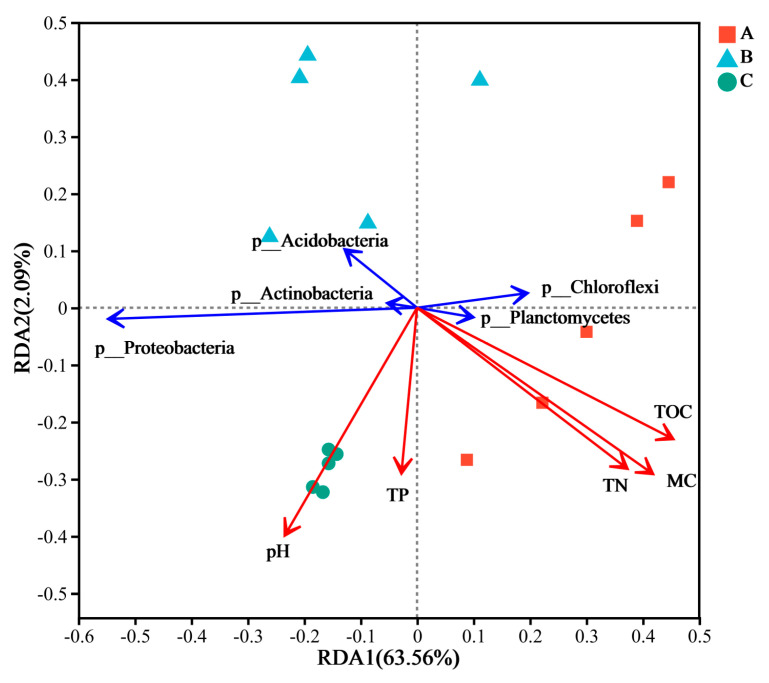
Redundancy analysis between environmental factors and bacterial communities in sediments (Sediment samples: red square A, blue triangle B, green circle C; arrows indicate the influence of environmental factors). A represents a natural river channel; B represents a river channel with ongoing large-scale sand mining activities; and C represents a river channel in which sand mining activities were terminated one year prior. The angle between sites and environmental factors indicates the direction of the correlation between them. The vertical projection was drawn from different samples, and the closer the projection point was, the greater the influence of the environmental factor on the sample was.

Species correlations between the physicochemical properties of the sediment and the bacterial community at the phylum classification level at the sample sites in different sand mining environments were analysed using redundancy analysis, as shown in Figure 7. The results revealed that the first axis (RDA1) and the second axis (RDA2) explained 62.47% and 2.12% of the variation in community structure at the microbial phylum level, respectively, and the cumulative amount of explained variation reached 64.59%, which reflects that sediment environmental factors have a certain influence on the structure of bacterial communities. Among microbial phyla, Proteobacteria and Actinobacteria were positively correlated with pH and TP content and negatively correlated with TOC, MC, and TN content, whereas Chloroflexi and Planctomycetes were positively correlated with TOC, MC, and TN content and negatively correlated with pH and TP content. Acidobacteria were negatively correlated with all five environmental factors, pH, TP, TN, MC, and TOC. In addition, the analytical structure also revealed that the TNt (r = 0.4785, *p* = 0.023), TOC (r = 0.5693, *p* = 0.007), and MC (r = 0.5112, *p* = 0.005) values at the sampling sites under the three different types of sand mining environments reached highly significant levels. In other words, the changes in TN, TOC, and MC caused by large-scale sand mining activities are the main limiting factors affecting the sediment bacterial community structure. In addition, the distance between the sample sites in the three sand mining environments and the scattered distribution of sites in the continuous sand mining channel and the channel where sand mining activities had been terminated one year prior, in addition to the clustered distribution of the sites in the natural channel, also suggest that sand mining affects the structure of the sediment bacterial community.

### 3.4. Functional Diversity of Sediment Bacterial Communities

Sediment bacterial communities in different sand mining environments were analysed in terms of primary and secondary functional metabolic pathways using R language software 2.0 prediction analysis and annotation by the KEGG database, as shown in Table 2 (The relevant data can be found in Supplementary Materials Tables S4–S6). There were six types of primary metabolic pathways in all the samples, and the primary metabolic pathways related to the sediment bacteria at the three sampling sites were metabolism, genetic information processing, environmental information processing, and cellular processes.

A total of 46 categories of pathways related to secondary metabolic functions were analysed at each sampling site, and 18 categories of secondary metabolic pathways (relative abundance of functional gene sequences > 1%) were related mainly to sediment bacteria at the three sampling sites. Among them, there were 11 types of secondary functions belonging to metabolism, three types of secondary functions belonging to genetic information processing, two belonging to environmental information processing, and two belonging to cellular processes; i.e., the primary metabolic pathway is dominated by metabolism. Sample site A had seven secondary functions that had a greater effect on the differential effect, with global and overview maps (27.75%) having the highest LDA score; sample site B had three secondary functions that had a greater effect on the differential effect, with exogenous biodegradation and metabolism (2.06%) having the highest LDA score; and sample site C had only one secondary function that had a greater effect on the differential effect.

Among the major functions of metabolism, the major secondary functions were carbohydrate metabolism (9.18%), amino acid metabolism (8.41%), and energy metabolism (7.55%). Compared with those of sampling site A, the carbohydrate metabolism function and energy metabolism function of sampling site B were significantly lower (*p* < 0.05), and its amino acid metabolism function was significantly greater than that of sampling site A (*p* < 0.05). Meanwhile, the carbohydrate metabolism function of sampling site C was significantly lower than that of sampling site A (*p* < 0.05), and its amino acid metabolism function and energy metabolism function were significantly greater than those of sampling site A (*p* < 0.05). The energy metabolism function was significantly greater (*p* < 0.05) at sampling site C than at sampling site B.

## 4. Discussion

### 4.1. Analysis of Differences in the Diversity of Sediment Bacterial Communities in Different Sand Mining Environments

The Jialing River is the basin with the largest sand content of all tributaries of the Yangtze River, and it is one of the key sand-mining rivers in the upper reaches of the Yangtze River. The diversity of bacterial communities in Jialing River sediments is an important indicator of the impact of sewage discharge, dam construction, and other activities on the water ecosystem and environmental quality of the basin [37,38]. With the development of urban construction, the demand for river sand has been increasing, leading to increasingly serious phenomena of indiscriminate mining [39]. Sand mining activities not only change river sediment and affect the distribution of suspended solids but also lead to changes in physicochemical properties and sediment bacterial communities among different habitats [40,41]. In this study, there were significant differences in the mean values of the ACE index among the three sites, with the greatest mean value at site A (no sand mining activities), followed by site B (currently impacted by sand mining activities) and site C (previously impacted by sand mining activities), while there were also different degrees of significant differences in the Shannon index and Simpson index among the three types of sampling sites. On the basis of the OTU counts of the sediment bacterial communities at the three sampling sites, the OTU counts of the sediments at sampling sites A were significantly greater than those at sampling sites B and C. In the vertical profile, the OTU counts of the deeper sediments were significantly lower than those of the surface sediments, and the OTU counts decreased and then increased with increasing sedimentation depth. These results indicate that large-scale sand mining activities significantly reduced the abundance of the sediment bacterial community (characterized by the number of OTUs) and had a significant effect on the bacterial community structure in both horizontal and vertical spatial dimensions. This phenomenon decreased and then increased with increasing depth, which indicated that large-scale sand mining activities significantly reduced the abundance of the sediment bacterial community and significantly affected the bacterial community both horizontally and vertically. This finding is broadly consistent with the results of studies examining the effects of sand mining on macroinvertebrates in large shallow lakes and the effects of river sand content on sediment bacterial communities [42,43]. The reason for this phenomenon may be that (i) large-scale sand mining activities excavate the river channel, thus destroying the silt layer; (ii) the silt layer collapses due to the hollowing out of the sand layer; (iii) the original surface sediment is covered, and the upstream sediment flows with the current to form new surface sediment, which results in a significant change in the vertical structure of the sediment; and (iv) it takes a time after the end of sand mining to re-establish a relatively stable sediment environment; i.e., changes in the diversity of the sediment bacterial community may be caused by the destruction of habitats that are suitable for native microorganisms by sand mining activities, and their survival is affected by changes in ecological niches, as well as by competition from foreign microorganisms. In addition, the reduction in sediment bacterial communities may be caused by large-scale instruments for sand mining releasing large amounts of metal elements into the aquatic environment during their use, resulting in the enrichment of metal elements in the aquatic ecosystem, which adversely affects the organisms in aquatic ecosystems, thus destroying the interaction between the bacterial community and aquatic organisms and ultimately leading to a reduction in the abundance of the sediment bacterial community [44].

In this study, the three dominant bacterial phyla in the sediments from three different sand mining environments were Proteobacteria, Chloroflexi, and Acidobacteria, with Proteobacteria dominating. This finding is similar to the findings of studies in which bacterial communities were used to monitor sediment contaminants, which revealed that the dominant phyla were Proteobacteria, Chloroflexi [45], and studies in which bacterial communities in subtropical rivers were affected by anthropogenic activities, which revealed that Proteobacteria exhibited strong adaptability in areas affected by sand mining [46]. The reason for this phenomenon may be that these dominant bacteria use facultative or obligate anaerobic Gram-negative bacteria as the main flora, which can effectively remove organic matter in anoxic water environments. In this study, the sediment TOC index values at sampling site B, which were affected by large-scale sand mining activities, tended to decrease compared with those at sampling site A, which was not subjected to sand mining activities, whereas the TOC index values at sampling site C, which was subjected to sand mining activities, tended to increase compared with those at sampling site B. This phenomenon is consistent with the changes in the relative abundances of Proteobacteria and Acidobacteria in the dominant groups of bacteria in the sediments of the three types of sampling sites. Proteobacteria can be metabolized by heterotrophic organisms or obtain energy from sunlight, and the fact that they have diverse energy pathways is conducive to them having flexibility in terms of suitable habitats [47]. During sand mining, the method and scale of sand mining may cause certain types of habitat destruction and metal pollution in the watershed, while the relevant literature shows that Proteobacteria contain resistance genes for most metals, which means that they play important roles in biological nitrogen and phosphorus removal and the degradation of pollutants [48]. This is consistent with the high TN content in this study; therefore, it is the main phylum in the sand mining environment. However, the composition of bacterial communities in different environments is specific to a certain extent. In this study, the relative abundance of Acidobacteria in the dominant phylum significantly decreased in the order of B, C, and A, whereas the relative abundance of Chloroflexi significantly decreased in the order of A, B, and C. Acidobacteria play important metabolic and biotransformation roles in one-carbon compounds in soil [49]. Large-scale sand mining activities change river sediment particle size, concentration, and the river benthic environment, thereby changing the bacterial community structure in the sediment. This further affects the functional properties of the bacterial communities, thus revealing the low abundance of Acidobacteria in the sampling sites affected by sand mining activity, with acidophilic properties. Although the pH in this study was weakly alkaline, the sediment pH affected by large-scale sand mining activities decreased compared with the sediment pH not disturbed by sand mining activities, leading to Acidobacteria becoming the main bacterial community composition in sediments disturbed by sand mining activities. This finding is consistent with the results of the sediment bacterial community in the Yellow River Basin [50]. The photosynthetic activity of Chloroflexi is an important driving factor of the carbon cycle. Sand mining activities change the vertical structure of sediments, resulting in the collapse of the silt layer to cover the original surface sediments, thus reducing photosynthesis and the abundance of Chloroflexi. Therefore, sand mining activities may further affect the carbon cycle process in sediments [51]. In addition, the processes of sand mining ships or sand mining machinery may cause oil leakage, and the oil floating on the surface of the water not only affects the light transmittance but also leads to a dissolved oxygen content in the water and Chloroflexi for aerobic bacteria, the aerobic metabolism pathway, so large-scale sand mining activities lead to a reduction in the abundance of Chloroflexi. The effects of sand mining activities on the composition of the sediment bacterial community may also be related to the redox process of S. In this study, one year after the termination of large-scale sand mining activities, there was a significant dominant strain of Desulfuromonadaceae in the sediments at sampling point C compared with those at the other two sampling points. Desulfuromonadaceae could reduce ferrous salt and sulfide contents to S and sulfate, and at the same time, bioremediation could be carried out to transform the pollutants in the soil and reduce the contents of chromium, iron, and other toxic metals in the soil, which was combined with the analysis of the physicochemical properties of the sediments. An analysis of the physical and chemical properties of the sediments, revealed that the S content in the sediments was in the order of A > C > B, and the S content in the sediments at the three sampling points tended to increase and then decrease with increasing depth, which indicated that large-scale sand mining activities inhibited the removal of Desulfuromonadaceae sulfuric acid; i.e., large-scale sand mining activities may have produced many ferrous salts and sulfides in the water body, which in turn, affected the structure of the bacterial community in the sediments [52].

The physical and chemical properties of sediments in different sand mining environments have important effects on the bacterial community structure in sediments. In this study, three environmental factors, TN, MC, and TOC, were the main limiting factors affecting the structure of sediment bacterial communities during large-scale sand mining activities. A study on the impact of mining on sediments in Dongting Lake revealed that pH, TOC, and TN significantly affect the bacterial community structure in sediments [53]. In addition, some studies have shown that the degree of phosphorus adsorption in river sediments is related to pH [54]. The cause of this phenomenon may be the direct or indirect transport of waste oil into the water, and large-scale sand mining activities disrupt the basin channel structure. In the river surface potholes, the upstream pollutants and the oil accumulation affect the carbon and nitrogen contents and the pH of the sediment in the basin, thus affecting the water environment and river environment, which in turn, affects Acidobacteria; this is consistent with previous studies showing that the sediment pH is weakly alkaline and that the TN content is high [55]. Studies on how artificial activities, such as sand mining, affect the elements of a basin have shown that N and P are dominant and that the TN content in the sediment bacterial community and the physical and chemical properties of the sediment significantly reduce the TN content in the sediment, which is not conducive to the survival of N as a nutrient source and may lead to changes in the bacterial community structure in the sediment [56]. In addition, studies have shown that sand mining activities significantly affect the sediment content of a basin, and a change in the sediment content in a river is the key environmental driver affecting the TOC, nitrogen, and phosphorus contents of the river [57], which is consistent with the results of this study; that is, a change in the TOC and TN contents of the sediment is the main limiting factor affecting the bacterial community structure of the sediment.

### 4.2. Functional Analysis of Sediment Bacteria in Different Sand Mining Environments

Soil microorganisms have catabolic functions that can change significantly under different disturbance environments [58,59]. With the development of high-throughput sequencing technology, in studies related to aquatic ecosystems, many researchers have used this technology to analyse the structural diversity and function of sediment bacterial communities affected by sewage discharge [60,61], and few studies have been conducted on the structure and function of sediment bacterial communities disturbed by sand mining activities. Therefore, PICRUSt functional prediction analysis was conducted on sediment bacteria in the natural river, in the channel with continuous sand mining, and in the channel in which sand mining had been terminated one year prior to provide a basis for the treatment of watersheds affected by sand mining activities. In this study, the major primary functions of the bacterial communities involved four categories, namely, metabolism, genetic information processing, environmental information processing, and cell processing. Among these categories, the relative abundance of metabolism was the highest, which may be correlated with the alkaline nature of the pH found in the physicochemical properties of the sediment in this study because the alkaline environment can promote the release of N, which results in the effective development of microbial metabolic pathways, and this finding is consistent with most related studies [62,63], suggesting that sand mining activities may mutate sediment bacterial communities to adapt to habitat changes. There were significant changes in 19 major functions of sediment bacteria in different sand mining environments, including global and synopsis functions, energy metabolism, cofactor and vitamin metabolism, translation, lipid metabolism and metabolism, folding, classification, and degradation. There were 7, 3, and 1 kind of secondary function (LDA > 3) in the sediment bacterial communities in the natural river, in the channel with continuous sand mining, and in the channel in which sand mining had been terminated one year prior, respectively. This finding indicates that large sand mining activities significantly inhibit most of the secondary functions of bacterial communities with a long influence cycle. Energy metabolism is the basis of all life activities. Its main process involves bacteria decomposing organic matter or oxidizing inorganic matter to release energy [64]. Carbohydrate metabolism regulates the formation, decomposition, and transformation of carbohydrates within organisms. The more vigorous the metabolic function is, the stronger the microbial life activity. This metabolic function is affected by seasonal changes and different environmental factors, among which TOC and TP are significantly affected [65]. In this study, compared with the secondary function of sediments in the natural river, the relative abundance of carbohydrate metabolism, energy metabolism, cofactor and vitamin metabolism, translation function, and nucleotide metabolism of sediments in the channel with continuous sand mining activities decreased significantly. The results indicated that sand mining activity inhibited the metabolism of carbohydrates and nucleotides and the replication and translation of genetic information from the sediment bacteria, which led to unfavourable variation in the bacterial community to adapt to changes in habitat and thus affected the structure and diversity of the bacterial community. However, because of the limitations of the database itself, the effects of different sand mining environments on the functioning of sediment bacterial communities can be further studied by combining macrogenome sequencing and other analytical tools.

## 5. Conclusions

The abundance of bacterial communities in different sand mining environments in the Jialing River Basin clearly affects the bacterial community structure. Large-scale sand mining activities lead to a significant decrease in the abundance of bacterial communities. Under different sand mining environments, the main dominant bacterial groups in the sediment of the Jialing River Basin were Proteobacteria, Chloroflexi, Acidobacteria, and Actinobacteria. Sustained sand mining activities increase the abundance of Proteobacteria and Acidobacteria but decrease the abundance of Chloroflexi. The contents of TOC, MC, and TN in sediments are the major driving forces affecting the composition and function of microbial communities. Continuous large-scale sand mining induces significant changes in major secondary functions, such as global and outline maps, energy metabolism, and cofactors. It also significantly inhibits carbohydrate metabolism, energy metabolism, cofactor and vitamin metabolism, translation, and nucleotide metabolism.

## Figures and Tables

**Table 1:** Alpha diversity index of the bacterial community in the sediments at each sampling point.

Sampling Point	Ace Index	Shannon Index	Simpson Index
A1	3397	4.28967	0.041277
A2	3380	4.131143	0.046858
A3	3382	4.356504	0.038633
A4	3309	4.457314	0.039228
A5	3400	4.399154	0.039693
B1	3268	4.311992	0.043663
B2	3167	4.380076	0.04315
B3	3076	4.311935	0.050867
B4	3071	4.310983	0.053499
B5	3255	4.505017	0.041089
C1	3009	4.648298	0.03563
C2	2902	4.511715	0.040251
C3	2927	4.651078	0.034217
C4	2944	4.634756	0.034542
C5	2982	4.613699	0.036462

Note: A represents a natural river channel; B represents a river channel with ongoing large-scale sand mining activities; and C represents a river channel in which sand mining activities were terminated one year prior.

**Table 2:** Relative abundance information of secondary functions of bacterial communities in sediments from different interference zones.

Secondary Function	A	B	C	Primary Function
global and overview maps	0.2755 ± 0.1852 ^b^	0.2717 ± 0.1122 ^b^	0.2693 ± 0.0056 ^c^	L1
carbohydrate metabolism	0.0947 ± 0.0026 ^a^	0.0899 ± 0.0019 ^b^	0.0908 ± 0.0006 ^b^	L1
amino acid metabolism	0.0804 ± 0.0017 ^b^	0.0854 ± 0.0029 ^a^	0.0866 ± 0.0007 ^a^	L1
energy metabolism	0.0751 ± 0.0005 ^b^	0.0730 ± 0.0019 ^a^	0.0785 ± 0.0004 ^c^	L1
metabolism of cofactors and vitamins	0.0480 ± 0.0007 ^a^	0.0460 ± 0.0007 ^b^	0.0444 ± 0.0007 ^c^	L1
membrane transport	0.0420 ± 0.0008 ^b^	0.0436 ± 0.0007 ^a^	0.0434 ± 0.0003 ^a^	L3
cellular community–prokaryotes	0.0390 ± 0.0005 ^a^	0.0392 ± 0.0007 ^a^	0.0392 ± 0.0002 ^a^	L4
signal transduction	0.0338 ± 0.0012 ^b^	0.0371 ± 0.0014 ^a^	0.0341 ± 0.0003 ^b^	L3
translation	0.0356 ± 0.0009 ^a^	0.0288 ± 0.0016 ^c^	0.0322 ± 0.0001 ^b^	L2
nucleotide metabolism	0.0291 ± 0.0004 ^a^	0.0282 ± 0.0006 ^b^	0.0292 ± 0.0001 ^a^	L1
replication and repair	0.0261 ± 0.0004 ^a^	0.0236 ± 0.0011 ^b^	0.0244 ± 0.0003 ^b^	L2
lipid metabolism	0.0229 ± 0.0002 ^c^	0.0245 ± 0.0005 ^a^	0.0236 ± 0.0003 ^b^	L1
metabolism of other amino acids	0.0207 ± 0.0002 ^c^	0.0241 ± 0.0013 ^a^	0.0226 ± 0.0002 ^b^	L1
folding, sorting and degradation	0.0201 ± 0.0004 ^b^	0.0194 ± 0.0005 ^c^	0.0216 ± 0.0003 ^a^	L2
xenobiotics biodegradation and metabolism	0.0157 ± 0.0008 ^b^	0.0206 ± 0.0021 ^a^	0.0198 ± 0.0041 ^a^	L1
glycan biosynthesis and metabolism	0.0183 ± 0.0014 ^a^	0.0163 ± 0.0011 ^b^	0.0139 ± 0.0003 ^c^	L1
biosynthesis of other secondary metabolites	0.0163 ± 0.0005 ^a^	0.0156 ± 0.0002 ^b^	0.0142 ± 0.0002 ^c^	L1
cell growth and death	0.0145 ± 0.0004 ^b^	0.0149 ± 0.0001 ^a^	0.0150 ± 0.0002 ^a^	L4
metabolism of terpenoids and polyketides	0.0130 ± 0.0013 ^a^	0.0120 ± 0.0002 ^a^	0.0115 ± 0.0001 ^b^	L1

Note: A represents a natural river channel; B represents a river channel with ongoing large-scale sand mining activities; and C represents a river channel in which sand mining activities were terminated one year prior. Different lowercase letters indicate significant differences between different sampling points (*p* ≤ 0.05), and the data in the table are presented as the means ± standard deviations (*n* = 5). L1–L4 are metabolism, genetic information processing, environmental information processing, and cellular processes of first-order functional pathways, respectively.

## Data Availability

The original contributions presented in this study are included in the article/Supplementary Material. Further inquiries can be directed to the corresponding author. The raw high-throughput sequencing data have been uploaded to the NCBI Sequence Read Archive database “https://www.ncbi.nlm.nih.gov/sra/ (accessed on 14 September 2024)” and are available under the SRA accession numbers SRR25569156, SRR25569200, and SRR25569600.

## Supplementary Materials

The following supporting information can be downloaded at: https://www.mdpi.com/article/10.3390/microorganisms13091998/s1, Table S1: Relative abundance of bacterial communities at various points along natural river channel A. Table S2: Relative abundance of bacterial communities at various points along natural river channel B. Table S3: Relative abundance of bacterial communities at various points along natural river channel C. Table S4: Relative abundance of secondary functions at various points along natural river channel A. Table S5: Relative abundance of secondary functions at various points along natural river channel B. Table S6: Relative abundance of secondary functions at various points along natural river channel C.
