# Supplementary material for: Driving Effects of Large-Scale Sand Mining Activities on Bacterial Communities in Subtropical River Sediments—A Case Study of the Jialing River
Source: Microorganisms. 2025 Aug 27;13(9):1998. doi: 10.3390/microorganisms13091998 (PMC12471679; doi:10.3390/microorganisms13091998)
Supplement: Supplementary file 1 [file microorganisms-13-01998-s001.zip › microorganisms-3802553-supplementary.pdf]

Supplementary materials

Table number: 6

Table S1. Relative abundance of bacterial communities at various points along natural river channel A.

#Taxonomy	A1	A2	A3	A4	A5
p__Proteobacteria;c__Deltaproteobacteria;o__unclassified_c__Deltaproteobacteria;f__unclassified_c__Deltaproteobacteria;g__unclassified_c__Deltaproteobacteria	0.1019	0.0966	0.1040	0.1266	0.1091
p__Acidobacteria;c__unclassified_p__Acidobacteria;o__unclassified_p__Acidobacteria;f__unclassified_p__Acidobacteria;g__unclassified_p__Acidobacteria	0.0519	0.0458	0.0482	0.0459	0.0527
p__Proteobacteria;c__Betaproteobacteria;o__unclassified_c__Betaproteobacteria;f__unclassified_c__Betaproteobacteria;g__unclassified_c__Betaproteobacteria	0.0080	0.0053	0.0164	0.0414	0.0241
p__Chloroflexi;c__unclassified_p__Chloroflexi;o__unclassified_p__Chloroflexi;f__unclassified_p__Chloroflexi;g__unclassified_p__Chloroflexi	0.1059	0.1088	0.0730	0.0576	0.0637
p__Proteobacteria;c__Gammaproteobacteria;o__Pseudomonadales;f__Pseudomonadaceae;g__Pseudomonas	0.0297	0.0270	0.0337	0.0552	0.0300
p__Candidatus_Aminicenantes;c__unclassified_p__Candidatus_Aminicenantes;o__unclassified_p__Candidatus_Aminicenantes;f__unclassified_p__Candidatus_Aminicenantes;g__unclassified_p__Candidatus_Aminicenantes	0.0450	0.0193	0.0210	0.0119	0.0914
p__Proteobacteria;c__Gammaproteobacteria;o__unclassified_c__Gammaproteobacteria;f__unclassified_c__Gammaproteobacteria;g__unclassified_c__Gammaproteobacteria	0.0036	0.0026	0.0043	0.0090	0.0107
p__unclassified_d__Bacteria;c__unclassified_d__Bacteria;o__unclassified_d__Bacteria;f__unclassified_d__Bacteria;g__unclassified_d__Bacteria	0.0304	0.0284	0.0288	0.0259	0.0275
p__Planctomycetes;c__unclassified_p__Planctomycetes;o__unclassified_p__Planctomycetes;f__unclassified_p__Planctomycetes;g__unclassified_p__Planctomycetes	0.0440	0.0480	0.0389	0.0216	0.0243
p__Gemmatimonadetes_d__Bacteria;c__unclassified_p__Gemmatimonadetes_d__Bacteria;o__unclassified_p__Gemmatimonadetes_d__Bacteria;f__unclassified_p__Gemmatimonadetes_d__Bacteria;g__unclassified_p__Gemmatimonadetes_d__Bacteria	0.0207	0.0404	0.0388	0.0140	0.0209
p__Spirochaetes;c__unclassified_p__Spirochaetes;o__unclassified_p__Spirochaetes;f__unclassified_p__Spirochaetes;g__unclassified_p__Spirochaetes	0.0293	0.0191	0.0249	0.0203	0.0214

p__Actinobacteria;c__Actinobacteria;o__unclassified_c__Actinobacteria;f__unclassified_c__Actinobacteria;g__unclassified_c__Actinobacteria	0.0062	0.0053	0.0068	0.0134	0.0100
p__Chloroflexi;c__Anaerolineae;o__unclassified_c__Anaerolineae;f__unclassified_c__Anaerolineae;g__unclassified_c__Anaerolineae	0.0352	0.0413	0.0175	0.0147	0.0180
p__Candidatus_Rokubacteria;c__unclassified_p__Candidatus_Rokubacteria;o__unclassified_p__Candidatus_Rokubacteria;f__unclassified_p__Candidatus_Rokubacteria;g__unclassified_p__Candidatus_Rokubacteria	0.0035	0.0031	0.0041	0.0082	0.0052
p__Proteobacteria;c__Alphaproteobacteria;o__Rhizobiales;f__Bradyrhizobiaceae;g__Bradyrhizobium	0.0011	0.0009	0.0010	0.0024	0.0013
p__Proteobacteria;c__Alphaproteobacteria;o__unclassified_c__Alphaproteobacteria;f__unclassified_c__Alphaproteobacteria;g__unclassified_c__Alphaproteobacteria	0.0044	0.0030	0.0033	0.0046	0.0034
p__Actinobacteria;c__unclassified_p__Actinobacteria;o__unclassified_p__Actinobacteria;f__unclassified_p__Actinobacteria;g__unclassified_p__Actinobacteria	0.0074	0.0071	0.0134	0.0063	0.0094
p__Proteobacteria;c__Deltaproteobacteria;o__Myxococcales;f__Anaeromyxobacteraceae;g__Anaeromyxobacter	0.0024	0.0014	0.0044	0.0048	0.0047
p__Nitrospirae;c__unclassified_p__Nitrospirae;o__unclassified_p__Nitrospirae;f__unclassified_p__Nitrospirae;g__unclassified_p__Nitrospirae	0.0091	0.0066	0.0074	0.0059	0.0055
p__Nitrospirae;c__Nitrospira;o__Nitrospirales;f__Nitrospiraceae;g__Nitrospira	0.0005	0.0006	0.0007	0.0015	0.0008
p__Proteobacteria;c__unclassified_p__Proteobacteria;o__unclassified_p__Proteobacteria;f__unclassified_p__Proteobacteria;g__unclassified_p__Proteobacteria	0.0033	0.0029	0.0034	0.0039	0.0032
p__Proteobacteria;c__Gammaproteobacteria;o__Pseudomonadales;f__Moraxellaceae;g__unclassified_f__Moraxellaceae	0.0020	0.0018	0.0014	0.0033	0.0016
p__Proteobacteria;c__Alphaproteobacteria;o__Rhizobiales;f__unclassified_o__Rhizobiales;g__unclassified_o__Rhizobiales	0.0079	0.0051	0.0050	0.0049	0.0044
p__Proteobacteria;c__Betaproteobacteria;o__Rhodocyclales;f__Azonexaceae;g__Dechloromonas	0.0025	0.0002	0.0159	0.0158	0.0113
p__Proteobacteria;c__Deltaproteobacteria;o__Myxococcales;f__unclassified_o__Myxococcales;g__unclassified_o__Myxococcales	0.0032	0.0044	0.0020	0.0027	0.0023

p__Candidatus_Abyssubacteria;c__unclassified_p__Candidatus_Abyssubacteria;o__unclassified_p__Candidatus_Abyssubacteria;f__unclassified_p__Candidatus_Abyssubacteria;g__unclassified_p__Candidatus_Abyssubacteria	0.0116	0.0271	0.0131	0.0150	0.0141
p__Proteobacteria;c__Deltaproteobacteria;o__Desulfobacterales;f__Desulfobacteraceae;g__unclassified_f__Desulfobacteraceae	0.0061	0.0044	0.0063	0.0115	0.0081
p__Proteobacteria;c__Deltaproteobacteria;o__Syntrophobacterales;f__Syntrophaceae;g__unclassified_f__Syntrophaceae	0.0035	0.0032	0.0036	0.0059	0.0026
p__Chloroflexi;c__Anaerolineae;o__Anaerolineales;f__unclassified_o__Anaerolineales;g__unclassified_o__Anaerolineales	0.0103	0.0100	0.0058	0.0038	0.0049
p__Verrucomicrobia;c__unclassified_p__Verrucomicrobia;o__unclassified_p__Verrucomicrobia;f__unclassified_p__Verrucomicrobia;g__unclassified_p__Verrucomicrobia	0.0048	0.0054	0.0041	0.0050	0.0043
p__candidate_division_Zixibacteria;c__unclassified_p__candidate_division_Zixibacteria;o__unclassified_p__candidate_division_Zixibacteria;f__unclassified_p__candidate_division_Zixibacteria;g__unclassified_p__candidate_division_Zixibacteria	0.0162	0.0121	0.0041	0.0103	0.0094
p__Proteobacteria;c__Betaproteobacteria;o__Burkholderiales;f__unclassified_o__Burkholderiales;g__unclassified_o__Burkholderiales	0.0007	0.0004	0.0011	0.0018	0.0013
p__Candidatus_Eisenbacteria;c__unclassified_p__Candidatus_Eisenbacteria;o__unclassified_p__Candidatus_Eisenbacteria;f__unclassified_p__Candidatus_Eisenbacteria;g__unclassified_p__Candidatus_Eisenbacteria	0.0015	0.0024	0.0012	0.0025	0.0020
p__Gemmatimonadetes_d__Bacteria;c__Gemmatimonadetes_p__Gemmatimonadetes;o__Gemmatimonadales;f__unclassified_o__Gemmatimonadales;g__unclassified_o__Gemmatimonadales	0.0028	0.0046	0.0044	0.0025	0.0031
p__Proteobacteria;c__Deltaproteobacteria;o__Desulfuromonadales;f__Desulfuromonadaceae;g__Desulfuromonas	0.0006	0.0007	0.0010	0.0019	0.0013
p__Proteobacteria;c__Deltaproteobacteria;o__Syntrophobacterales;f__unclassified_o__Syntrophobacterales;g__unclassified_o__Syntrophobacterales	0.0015	0.0013	0.0016	0.0019	0.0014
p__Proteobacteria;c__Alphaproteobacteria;o__Rhodospirillales;f__Rhodospirillaceae;g__unclassified_f__Rhodospirillaceae	0.0005	0.0004	0.0006	0.0016	0.0009

p__Chloroflexi;c__Anaerolineae;o__Anaerolineales;f__Anaerolineaceae;g__unclassified_f__Anaerolineaceae	0.0062	0.0070	0.0042	0.0036	0.0040
p__Planctomycetes;c__Phycisphaerae;o__unclassified_c__Phycisphaerae;f__unclassified_c__Phycisphaerae;g__unclassified_c__Phycisphaerae	0.0079	0.0086	0.0080	0.0046	0.0049
p__Chloroflexi;c__Dehalococcoidia;o__unclassified_c__Dehalococcoidia;f__unclassified_c__Dehalococcoidia;g__unclassified_c__Dehalococcoidia	0.0080	0.0060	0.0062	0.0029	0.0044
p__Nitrospirae;c__Nitrospira;o__Nitrospirales;f__Nitrospiraceae;g__unclassified_f__Nitrospiraceae	0.0015	0.0013	0.0014	0.0017	0.0014
p__Proteobacteria;c__Betaproteobacteria;o__Nitrosomonadales;f__unclassified_o__Nitrosomonadales;g__unclassified_o__Nitrosomonadales	0.0002	0.0002	0.0004	0.0008	0.0011
p__candidate_division_NC10;c__unclassified_p__candidate_division_NC10;o__unclassified_p__candidate_division_NC10;f__unclassified_p__candidate_division_NC10;g__unclassified_p__candidate_division_NC10	0.0030	0.0022	0.0022	0.0022	0.0021
p__Acidobacteria;c__Thermoanaerobaculia;o__unclassified_c__Thermoanaerobaculia;f__unclassified_c__Thermoanaerobaculia;g__unclassified_c__Thermoanaerobaculia	0.0006	0.0007	0.0006	0.0009	0.0010
p__Proteobacteria;c__Betaproteobacteria;o__Nitrosomonadales;f__Thiobacillaceae;g__Thiobacillus	0.0004	0.0001	0.0023	0.0038	0.0123
p__Proteobacteria;c__Betaproteobacteria;o__Rhodocyclales;f__Rhodocyclaceae;g__Aromatoleum	0.0002	0.0002	0.0004	0.0009	0.0006
p__Bacteroidetes;c__unclassified_p__Bacteroidetes;o__unclassified_p__Bacteroidetes;f__unclassified_p__Bacteroidetes;g__unclassified_p__Bacteroidetes	0.0032	0.0032	0.0039	0.0046	0.0039
p__Proteobacteria;c__Betaproteobacteria;o__Burkholderiales;f__Burkholderiaceae;g__unclassified_f__Burkholderiaceae	0.0004	0.0002	0.0005	0.0010	0.0007
p__Proteobacteria;c__Gammaproteobacteria;o__Xanthomonadales;f__unclassified_o__Xanthomonadales;g__unclassified_o__Xanthomonadales	0.0006	0.0004	0.0008	0.0016	0.0011
p__Proteobacteria;c__Deltaproteobacteria;o__Desulfobacterales;f__unclassified_o__Desulfobacterales;g__unclassified_o__Desulfobacterales	0.0047	0.0031	0.0053	0.0037	0.0036
p__Proteobacteria;c__Deltaproteobacteria;o__Syntrophobacterales;f__Syntrophaceae;g__Syntrophus_f__Syntrophaceae	0.0010	0.0011	0.0013	0.0014	0.0012

p__Planctomycetes;c__Phycisphaerae;o__Phycisphaerales;f__unclassified_o__Phycisphaerales;g__unclassified_o__Phycisphaerales	0.0066	0.0069	0.0087	0.0032	0.0037
p__Proteobacteria;c__Betaproteobacteria;o__Rhodocyclales;f__Rhodocyclaceae;g__unclassified_f__Rhodocyclaceae	0.0005	0.0002	0.0019	0.0027	0.0019
p__Proteobacteria;c__Gammaproteobacteria;o__Xanthomonadales;f__Xanthomonadaceae;g__Stenotrophomonas	0.0032	0.0035	0.0033	0.0046	0.0036
p__Acidobacteria;c__Vicinamibacteria;o__unclassified_c__Vicinamibacteria;f__Vicinamibacteraceae;g__Luteitalea	0.0014	0.0015	0.0013	0.0015	0.0016
p__Planctomycetes;c__Planctomycetia;o__Planctomycetales;f__Planctomycetaceae;g__unclassified_f__Planctomycetaceae	0.0049	0.0054	0.0050	0.0030	0.0032
p__Proteobacteria;c__Deltaproteobacteria;o__Desulfuromonadales;f__Geobacteraceae;g__Geobacter	0.0014	0.0013	0.0019	0.0031	0.0023
p__Proteobacteria;c__Gammaproteobacteria;o__Acidiferrobacterales;f__Acidiferrobacteraceae;g__Sulfuricaulis	0.0001	0.0000	0.0001	0.0004	0.0002
p__Proteobacteria;c__Alphaproteobacteria;o__Rhizobiales;f__Xanthobacteraceae;g__Pseudolabrys	0.0003	0.0002	0.0006	0.0021	0.0010
p__Acidobacteria;c__Holophagae;o__unclassified_c__Holophagae;f__unclassified_c__Holophagae;g__unclassified_c__Holophagae	0.0025	0.0025	0.0016	0.0018	0.0021
p__Acidobacteria;c__Acidobacteriia;o__Bryobacterales;f__Solibacteraceae;g__Candidatus_Solibacter	0.0035	0.0035	0.0021	0.0020	0.0022
p__Proteobacteria;c__Zetaproteobacteria;o__unclassified_c__Zetaproteobacteria;f__unclassified_c__Zetaproteobacteria;g__unclassified_c__Zetaproteobacteria	0.0022	0.0013	0.0013	0.0012	0.0011
p__Proteobacteria;c__Alphaproteobacteria;o__Sphingomonadales;f__Sphingomonadaceae;g__Novosphingobium	0.0001	0.0001	0.0002	0.0006	0.0002
p__Proteobacteria;c__Alphaproteobacteria;o__Rhizobiales;f__Hyphomicrobiaceae;g__Rhodoplanes	0.0002	0.0002	0.0003	0.0006	0.0004
p__Proteobacteria;c__Alphaproteobacteria;o__Rhodospirillales;f__unclassified_o__Rhodospirillales;g__unclassified_o__Rhodospirillales	0.0003	0.0002	0.0003	0.0006	0.0004

p__Proteobacteria;c__Betaproteobacteria;o__Burkholderiales;f__unclassified_o__Burkholderiales;g__Piscinibacter	0.0001	0.0000	0.0003	0.0006	0.0003
p__Verrucomicrobia;c__Verrucomicrobiae;o__Verrucomicrobiales;f__Verrucomicrobia_subdivision_3;g__unclassified_f__Verrucomicrobia_subdivision_3	0.0020	0.0023	0.0017	0.0017	0.0017
p__Proteobacteria;c__Betaproteobacteria;o__Burkholderiales;f__unclassified_o__Burkholderiales;g__Rubrivivax	0.0002	0.0000	0.0004	0.0007	0.0004
p__Proteobacteria;c__Betaproteobacteria;o__Burkholderiales;f__Burkholderiaceae;g__Ralstonia	0.0031	0.0032	0.0032	0.0032	0.0034
p__Proteobacteria;c__Betaproteobacteria;o__Burkholderiales;f__Comamonadaceae;g__unclassified_f__Comamonadaceae	0.0002	0.0001	0.0003	0.0006	0.0004
p__Proteobacteria;c__Betaproteobacteria;o__Burkholderiales;f__Comamonadaceae;g__Ramlibacter	0.0002	0.0002	0.0003	0.0006	0.0005
p__Proteobacteria;c__Candidatus_Muproteobacteria;o__unclassified_c__Candidatus_Muproteobacteria;f__unclassified_c__Candidatus_Muproteobacteria;g__unclassified_c__Candidatus_Muproteobacteria	0.0002	0.0001	0.0002	0.0004	0.0003
p__Proteobacteria;c__Betaproteobacteria;o__Burkholderiales;f__unclassified_o__Burkholderiales;g__Ideonella	0.0001	0.0000	0.0003	0.0006	0.0004
p__Firmicutes;c__unclassified_p__Firmicutes;o__unclassified_p__Firmicutes;f__unclassified_p__Firmicutes;g__unclassified_p__Firmicutes	0.0033	0.0028	0.0030	0.0020	0.0022
p__Candidatus_Omnitrophica;c__unclassified_p__Candidatus_Omnitrophica;o__unclassified_p__Candidatus_Omnitrophica;f__unclassified_p__Candidatus_Omnitrophica;g__unclassified_p__Candidatus_Omnitrophica	0.0024	0.0041	0.0075	0.0013	0.0021
p__Armatimonadetes;c__unclassified_p__Armatimonadetes;o__unclassified_p__Armatimonadetes;f__unclassified_p__Armatimonadetes;g__unclassified_p__Armatimonadetes	0.0030	0.0030	0.0024	0.0016	0.0017
p__Proteobacteria;c__Alphaproteobacteria;o__Rhizobiales;f__Xanthobacteraceae;g__unclassified_f__Xanthobacteraceae	0.0002	0.0001	0.0002	0.0005	0.0004
p__Proteobacteria;c__Deltaproteobacteria;o__Syntrophobacterales;f__Syntrophaceae;g__Desulfobacca	0.0034	0.0017	0.0017	0.0013	0.0015
p__Proteobacteria;c__Gammaproteobacteria;o__Nevskiales;f__Steroidobacteraceae;g__Steroidobacter	0.0001	0.0001	0.0002	0.0004	0.0002

p__Proteobacteria;c__Hydrogenophilalia;o__Hydrogenophilales;f__unclassified_o__Hydrogenophilales;g__unclassified_o__Hydrogenophilales	0.0002	0.0001	0.0010	0.0018	0.0050
p__Acidobacteria;c__Blastocatellia;o__unclassified_c__Blastocatellia;f__unclassified_c__Blastocatellia;g__unclassified_c__Blastocatellia	0.0013	0.0013	0.0011	0.0009	0.0013
p__Spirochaetes;c__Spirochaetia;o__Spirochaetales;f__unclassified_o__Spirochaetales;g__unclassified_o__Spirochaetales	0.0028	0.0020	0.0025	0.0023	0.0021
p__Proteobacteria;c__Gammaproteobacteria;o__Thiotrichales;f__Thiotrichaceae;g__Thioploca	0.0001	0.0001	0.0000	0.0001	0.0001
p__Proteobacteria;c__Betaproteobacteria;o__Burkholderiales;f__Comamonadaceae;g__Variovorax	0.0002	0.0002	0.0003	0.0005	0.0004
p__Elusimicrobia;c__unclassified_p__Elusimicrobia;o__unclassified_p__Elusimicrobia;f__unclassified_p__Elusimicrobia;g__unclassified_p__Elusimicrobia	0.0016	0.0039	0.0019	0.0009	0.0013
p__Actinobacteria;c__Actinobacteria;o__Corynebacteriales;f__Nocardiaceae;g__Rhodococcus_f__Nocardiaceae	0.0019	0.0017	0.0013	0.0026	0.0022
p__candidate_division_NC10;c__unclassified_p__candidate_division_NC10;o__unclassified_p__candidate_division_NC10;f__unclassified_p__candidate_division_NC10;g__Candidatus_Methylomirabilis	0.0003	0.0003	0.0003	0.0002	0.0002
p__Proteobacteria;c__Betaproteobacteria;o__Rhodocyclales;f__unclassified_o__Rhodocyclales;g__unclassified_o__Rhodocyclales	0.0002	0.0001	0.0006	0.0007	0.0005
p__Proteobacteria;c__Alphaproteobacteria;o__Sphingomonadales;f__Sphingomonadaceae;g__Sphingomonas	0.0002	0.0002	0.0002	0.0004	0.0003
p__Actinobacteria;c__Actinobacteria;o__Propionibacteriales;f__Propionibacteriaceae;g__Brooklawnia	0.0007	0.0000	0.0014	0.0105	0.0058
p__Cyanobacteria;c__unclassified_p__Cyanobacteria;o__Oscillatoriales;f__Microcoleaceae;g__unclassified_f__Microcoleaceae	0.0027	0.0001	0.0121	0.0015	0.0019
p__Acidobacteria;c__Acidobacteriia;o__Bryobacterales;f__unclassified_o__Bryobacterales;g__unclassified_o__Bryobacterales	0.0010	0.0010	0.0008	0.0008	0.0008
p__Chrysiogenetes_d__Bacteria;c__Chrysiogenetes_p__Chrysiogenetes;o__Chrysiogenales;f__unclassified_o__Chrysiogenales;g__unclassified_o__Chrysiogenales	0.0017	0.0009	0.0008	0.0014	0.0048

p__Bacteroidetes;c__Bacteroidia;o__Bacteroidales;f__unclassified_o__Bacteroidales;g__unclassified_o__Bacteroidales	0.0010	0.0004	0.0014	0.0061	0.0026
p__Proteobacteria;c__Alphaproteobacteria;o__Rhizobiales;f__Hyphomicrobiaceae;g__Hyphomicrobium	0.0010	0.0006	0.0006	0.0008	0.0006
p__Actinobacteria;c__Rubrobacteria;o__Gaiellales;f__Gaiellaceae;g__Gaiella	0.0002	0.0001	0.0002	0.0007	0.0003
p__Lentisphaerae;c__unclassified_p__Lentisphaerae;o__unclassified_p__Lentisphaerae;f__unclassified_p__Lentisphaerae;g__unclassified_p__Lentisphaerae	0.0022	0.0020	0.0017	0.0013	0.0014
p__Proteobacteria;c__Betaproteobacteria;o__Burkholderiales;f__unclassified_o__Burkholderiales;g__Methylibium	0.0001	0.0001	0.0002	0.0004	0.0002
p__Proteobacteria;c__Betaproteobacteria;o__Burkholderiales;f__Comamonadaceae;g__Curvibacter	0.0001	0.0000	0.0002	0.0005	0.0016
p__Gemmatimonadetes_d__Bacteria;c__Gemmatimonadetes_p__Gemmatimonadetes;o__Gemmatimonadales;f__Gemmatimonadaceae;g__Gemmatimonas	0.0011	0.0019	0.0019	0.0008	0.0012
p__Actinobacteria;c__Actinobacteria;o__Propionibacteriales;f__Nocardioidaceae;g__Nocardioides	0.0006	0.0003	0.0003	0.0008	0.0006
p__Candidatus_Latescibacteria;c__unclassified_p__Candidatus_Latescibacteria;o__unclassified_p__Candidatus_Latescibacteria;f__unclassified_p__Candidatus_Latescibacteria;g__unclassified_p__Candidatus_Latescibacteria	0.0018	0.0017	0.0014	0.0011	0.0014
p__Chloroflexi;c__Thermoflexia;o__unclassified_c__Thermoflexia;f__unclassified_c__Thermoflexia;g__unclassified_c__Thermoflexia	0.0044	0.0053	0.0013	0.0012	0.0019
p__Proteobacteria;c__Gammaproteobacteria;o__Thiotrichales;f__Thiotrichaceae;g__Beggiatoa	0.0000	0.0000	0.0000	0.0000	0.0001
p__Candidatus_Woesebacteria;c__unclassified_p__Candidatus_Woesebacteria;o__unclassified_p__Candidatus_Woesebacteria;f__unclassified_p__Candidatus_Woesebacteria;g__unclassified_p__Candidatus_Woesebacteria	0.0027	0.0009	0.0069	0.0005	0.0006
p__Candidatus_Bipolaricaulota;c__unclassified_p__Candidatus_Bipolaricaulota;o__unclassified_p__Candidatus_Bipolaricaulota;f__unclassified_p__Candidatus_Bipolaricaulota;g__unclassified_p__Candidatus_Bipolaricaulota	0.0037	0.0008	0.0029	0.0002	0.0005
other	0.2634	0.2982	0.3034	0.2990	0.2685

Table S2. Relative abundance of bacterial communities at various points along natural river channel B.

#Taxonomy	B1	B2	B3	B4	B5
p__Proteobacteria;c__Deltaproteobacteria;o__unclassified_c__Deltaproteobacteria;f__unclassified_c__Deltaproteobacteria;g__unclassified_c__Deltaproteobacteria	0.0873	0.0859	0.0693	0.0708	0.0549
p__Acidobacteria;c__unclassified_p__Acidobacteria;o__unclassified_p__Acidobacteria;f__unclassified_p__Acidobacteria;g__unclassified_p__Acidobacteria	0.1003	0.0989	0.1352	0.1409	0.1163
p__Proteobacteria;c__Betaproteobacteria;o__unclassified_c__Betaproteobacteria;f__unclassified_c__Betaproteobacteria;g__unclassified_c__Betaproteobacteria	0.0585	0.1132	0.1284	0.1340	0.0969
p__Chloroflexi;c__unclassified_p__Chloroflexi;o__unclassified_p__Chloroflexi;f__unclassified_p__Chloroflexi;g__unclassified_p__Chloroflexi	0.1031	0.0727	0.0481	0.0440	0.0367
p__Proteobacteria;c__Gammaproteobacteria;o__Pseudomonadales;f__Pseudomonadaceae;g__Pseudomonas	0.0507	0.0282	0.0223	0.0147	0.0130
p__Candidatus_Aminicenantes;c__unclassified_p__Candidatus_Aminicenantes;o__unclassified_p__Candidatus_Aminicenantes;f__unclassified_p__Candidatus_Aminicenantes;g__unclassified_p__Candidatus_Aminicenantes	0.0497	0.0190	0.0030	0.0026	0.0022
p__Proteobacteria;c__Gammaproteobacteria;o__unclassified_c__Gammaproteobacteria;f__unclassified_c__Gammaproteobacteria;g__unclassified_c__Gammaproteobacteria	0.0105	0.0234	0.0394	0.0356	0.0383
p__unclassified_d__Bacteria;c__unclassified_d__Bacteria;o__unclassified_d__Bacteria;f__unclassified_d__Bacteria;g__unclassified_d__Bacteria	0.0288	0.0192	0.0112	0.0109	0.0110
p__Planctomycetes;c__unclassified_p__Planctomycetes;o__unclassified_p__Planctomycetes;f__unclassified_p__Planctomycetes;g__unclassified_p__Planctomycetes	0.0121	0.0105	0.0063	0.0066	0.0063
p__Gemmatimonadetes_d__Bacteria;c__unclassified_p__Gemmatimonadetes_d__Bacteria;o__unclassified_p__Gemmatimonadetes_d__Bacteria;f__unclassified_p__Gemmatimonadetes_d__Bacteria;g__unclassified_p__Gemmatimonadetes_d__Bacteria	0.0125	0.0156	0.0146	0.0150	0.0105
p__Spirochaetes;c__unclassified_p__Spirochaetes;o__unclassified_p__Spirochaetes;f__unclassified_p__Spir	0.0168	0.0117	0.0036	0.0035	0.0027

ochaetes;g__unclassified_p__Spirochaetes					
p__Actinobacteria;c__Actinobacteria;o__unclassified_c__Actinobacteria;f__unclassified_c__Actinobacteria;g__unclassified_c__Actinobacteria	0.0177	0.0152	0.0196	0.0192	0.0202
p__Chloroflexi;c__Anaerolineae;o__unclassified_c__Anaerolineae;f__unclassified_c__Anaerolineae;g__unclassified_c__Anaerolineae	0.0199	0.0126	0.0051	0.0055	0.0051
p__Candidatus_Rokubacteria;c__unclassified_p__Candidatus_Rokubacteria;o__unclassified_p__Candidatus_Rokubacteria;f__unclassified_p__Candidatus_Rokubacteria;g__unclassified_p__Candidatus_Rokubacteria	0.0075	0.0181	0.0127	0.0111	0.0083
p__Proteobacteria;c__Alphaproteobacteria;o__Rhizobiales;f__Bradyrhizobiaceae;g__Bradyrhizobium	0.0022	0.0048	0.0232	0.0167	0.0663
p__Proteobacteria;c__Alphaproteobacteria;o__unclassified_c__Alphaproteobacteria;f__unclassified_c__Alphaproteobacteria;g__unclassified_c__Alphaproteobacteria	0.0045	0.0100	0.0170	0.0171	0.0289
p__Actinobacteria;c__unclassified_p__Actinobacteria;o__unclassified_p__Actinobacteria;f__unclassified_p__Actinobacteria;g__unclassified_p__Actinobacteria	0.0191	0.0101	0.0058	0.0054	0.0045
p__Proteobacteria;c__Deltaproteobacteria;o__Myxococcales;f__Anaeromyxobacteraceae;g__Anaeromyxobacter	0.0032	0.0064	0.0215	0.0281	0.0329
p__Nitrospirae;c__unclassified_p__Nitrospirae;o__unclassified_p__Nitrospirae;f__unclassified_p__Nitrospirae;g__unclassified_p__Nitrospirae	0.0137	0.0133	0.0105	0.0105	0.0076
p__Nitrospirae;c__Nitrospira;o__Nitrospirales;f__Nitrospiraceae;g__Nitrospira	0.0011	0.0092	0.0150	0.0158	0.0102
p__Proteobacteria;c__unclassified_p__Proteobacteria;o__unclassified_p__Proteobacteria;f__unclassified_p__Proteobacteria;g__unclassified_p__Proteobacteria	0.0046	0.0069	0.0116	0.0099	0.0218
p__Proteobacteria;c__Gammaproteobacteria;o__Pseudomonadales;f__Moraxellaceae;g__unclassified_f__Moraxellaceae	0.0007	0.0010	0.0001	0.0000	0.0001
p__Proteobacteria;c__Alphaproteobacteria;o__Rhizobiales;f__unclassified_o__Rhizobiales;g__unclassified_o__Rhizobiales	0.0049	0.0061	0.0106	0.0110	0.0093
p__Proteobacteria;c__Betaproteobacteria;o__Rhodocyclales;f__Azonexaceae;g__Dechloromonas	0.0004	0.0008	0.0016	0.0017	0.0010

p__Proteobacteria;c__Deltaproteobacteria;o__Myxococcales;f__unclassified_o__Myxococcales;g__unclassified_o__Myxococcales	0.0034	0.0078	0.0076	0.0087	0.0103
p__Candidatus_Abyssubacteria;c__unclassified_p__Candidatus_Abyssubacteria;o__unclassified_p__Candidatus_Abyssubacteria;f__unclassified_p__Candidatus_Abyssubacteria;g__unclassified_p__Candidatus_Abyssubacteria	0.0003	0.0003	0.0001	0.0001	0.0001
p__Proteobacteria;c__Deltaproteobacteria;o__Desulfobacterales;f__Desulfobacteraceae;g__unclassified_f__Desulfobacteraceae	0.0060	0.0042	0.0032	0.0030	0.0033
p__Proteobacteria;c__Deltaproteobacteria;o__Syntrophobacterales;f__Syntrophaceae;g__unclassified_f__Syntrophaceae	0.0050	0.0035	0.0009	0.0008	0.0007
p__Chloroflexi;c__Anaerolineae;o__Anaerolineales;f__unclassified_o__Anaerolineales;g__unclassified_o__Anaerolineales	0.0093	0.0059	0.0023	0.0023	0.0019
p__Verrucomicrobia;c__unclassified_p__Verrucomicrobia;o__unclassified_p__Verrucomicrobia;f__unclassified_p__Verrucomicrobia;g__unclassified_p__Verrucomicrobia	0.0036	0.0039	0.0031	0.0033	0.0033
p__candidate_division_Zixibacteria;c__unclassified_p__candidate_division_Zixibacteria;o__unclassified_p__candidate_division_Zixibacteria;f__unclassified_p__candidate_division_Zixibacteria;g__unclassified_p__candidate_division_Zixibacteria	0.0041	0.0024	0.0006	0.0007	0.0007
p__Proteobacteria;c__Betaproteobacteria;o__Burkholderiales;f__unclassified_o__Burkholderiales;g__unclassified_o__Burkholderiales	0.0051	0.0042	0.0040	0.0039	0.0036
p__Candidatus_Eisenbacteria;c__unclassified_p__Candidatus_Eisenbacteria;o__unclassified_p__Candidatus_Eisenbacteria;f__unclassified_p__Candidatus_Eisenbacteria;g__unclassified_p__Candidatus_Eisenbacteria	0.0045	0.0040	0.0046	0.0047	0.0035
p__Gemmatimonadetes_d__Bacteria;c__Gemmatimonadetes_p__Gemmatimonadetes;o__Gemmatimonadales;f__unclassified_o__Gemmatimonadales;g__unclassified_o__Gemmatimonadales	0.0029	0.0032	0.0037	0.0042	0.0032
p__Proteobacteria;c__Deltaproteobacteria;o__Desulfuromonadales;f__Desulfuromonadaceae;g__Desulfuromonas	0.0012	0.0012	0.0009	0.0010	0.0006
p__Proteobacteria;c__Deltaproteobacteria;o__Syntrophobacterales;f__unclassified_o__Syntrophobacterales;	0.0025	0.0018	0.0015	0.0014	0.0014

g__unclassified_o__Syntrophobacterales					
p__Proteobacteria;c__Alphaproteobacteria;o__Rhodospirillales;f__Rhodospirillaceae;g__unclassified_f__Rhodospirillaceae	0.0016	0.0055	0.0098	0.0069	0.0070
p__Chloroflexi;c__Anaerolineae;o__Anaerolineales;f__Anaerolineaceae;g__unclassified_f__Anaerolineaceae	0.0056	0.0034	0.0010	0.0011	0.0011
p__Planctomycetes;c__Phycisphaerae;o__unclassified_c__Phycisphaerae;f__unclassified_c__Phycisphaerae;g__unclassified_c__Phycisphaerae	0.0027	0.0023	0.0013	0.0013	0.0019
p__Chloroflexi;c__Dehalococcoidia;o__unclassified_c__Dehalococcoidia;f__unclassified_c__Dehalococcoidia;g__unclassified_c__Dehalococcoidia	0.0071	0.0032	0.0014	0.0014	0.0012
p__Nitrospirae;c__Nitrospira;o__Nitrospirales;f__Nitrospiraceae;g__unclassified_f__Nitrospiraceae	0.0030	0.0055	0.0071	0.0076	0.0050
p__Proteobacteria;c__Betaproteobacteria;o__Nitrosomonadales;f__unclassified_o__Nitrosomonadales;g__unclassified_o__Nitrosomonadales	0.0067	0.0187	0.0026	0.0033	0.0021
p__candidate_division_NC10;c__unclassified_p__candidate_division_NC10;o__unclassified_p__candidate_division_NC10;f__unclassified_p__candidate_division_NC10;g__unclassified_p__candidate_division_NC10	0.0037	0.0034	0.0021	0.0023	0.0015
p__Acidobacteria;c__Thermoanaerobaculia;o__unclassified_c__Thermoanaerobaculia;f__unclassified_c__Thermoanaerobaculia;g__unclassified_c__Thermoanaerobaculia	0.0018	0.0036	0.0109	0.0083	0.0076
p__Proteobacteria;c__Betaproteobacteria;o__Nitrosomonadales;f__Thiobacillaceae;g__Thiobacillus	0.0079	0.0023	0.0021	0.0021	0.0018
p__Proteobacteria;c__Betaproteobacteria;o__Rhodocyclales;f__Rhodocyclaceae;g__Aromatoleum	0.0024	0.0028	0.0043	0.0032	0.0032
p__Bacteroidetes;c__unclassified_p__Bacteroidetes;o__unclassified_p__Bacteroidetes;f__unclassified_p__Bacteroidetes;g__unclassified_p__Bacteroidetes	0.0041	0.0026	0.0017	0.0021	0.0019
p__Proteobacteria;c__Betaproteobacteria;o__Burkholderiales;f__Burkholderiaceae;g__unclassified_f__Burkholderiaceae	0.0027	0.0059	0.0049	0.0048	0.0036
p__Proteobacteria;c__Gammaproteobacteria;o__Xanthomonadales;f__unclassified_o__Xanthomonadales;g__unclassified_o__Xanthomonadales	0.0017	0.0023	0.0040	0.0040	0.0036

p__Proteobacteria;c__Deltaproteobacteria;o__Desulfobacterales;f__unclassified_o__Desulfobacterales;g__unclassified_o__Desulfobacterales	0.0044	0.0027	0.0012	0.0011	0.0010
p__Proteobacteria;c__Deltaproteobacteria;o__Syntrophobacterales;f__Syntrophaceae;g__Syntrophus_f__Syntrophaceae	0.0015	0.0012	0.0005	0.0006	0.0005
p__Planctomycetes;c__Phycisphaerae;o__Phycisphaerales;f__unclassified_o__Phycisphaerales;g__unclassified_o__Phycisphaerales	0.0011	0.0012	0.0004	0.0005	0.0005
p__Proteobacteria;c__Betaproteobacteria;o__Rhodocyclales;f__Rhodocyclaceae;g__unclassified_f__Rhodocyclaceae	0.0010	0.0016	0.0018	0.0016	0.0013
p__Proteobacteria;c__Gammaproteobacteria;o__Xanthomonadales;f__Xanthomonadaceae;g__Stenotrophomonas	0.0042	0.0019	0.0015	0.0010	0.0009
p__Acidobacteria;c__Vicinamibacteria;o__unclassified_c__Vicinamibacteria;f__Vicinamibacteraceae;g__Luteitalea	0.0035	0.0031	0.0038	0.0040	0.0033
p__Planctomycetes;c__Planctomycetia;o__Planctomycetales;f__Planctomycetaceae;g__unclassified_f__Planctomycetaceae	0.0020	0.0016	0.0010	0.0010	0.0010
p__Proteobacteria;c__Deltaproteobacteria;o__Desulfuromonadales;f__Geobacteraceae;g__Geobacter	0.0024	0.0015	0.0022	0.0041	0.0016
p__Proteobacteria;c__Gammaproteobacteria;o__Acidiferrobacterales;f__Acidiferrobacteraceae;g__Sulfuricaulis	0.0032	0.0065	0.0072	0.0070	0.0061
p__Proteobacteria;c__Alphaproteobacteria;o__Rhizobiales;f__Xanthobacteraceae;g__Pseudolabrys	0.0015	0.0026	0.0040	0.0039	0.0037
p__Acidobacteria;c__Holophagae;o__unclassified_c__Holophagae;f__unclassified_c__Holophagae;g__unclassified_c__Holophagae	0.0033	0.0022	0.0017	0.0015	0.0013
p__Acidobacteria;c__Acidobacteriia;o__Bryobacterales;f__Solibacteraceae;g__Candidatus_Solibacter	0.0028	0.0022	0.0013	0.0012	0.0011
p__Proteobacteria;c__Zetaproteobacteria;o__unclassified_c__Zetaproteobacteria;f__unclassified_c__Zetaproteobacteria;g__unclassified_c__Zetaproteobacteria	0.0028	0.0025	0.0014	0.0016	0.0011
p__Proteobacteria;c__Alphaproteobacteria;o__Sphingomonadales;f__Sphingomonadaceae;g__Novosphingobium	0.0002	0.0004	0.0025	0.0009	0.0009

p__Proteobacteria;c__Alphaproteobacteria;o__Rhizobiales;f__Hyphomicrobiaceae;g__Rhodoplanes	0.0009	0.0019	0.0062	0.0060	0.0060
p__Proteobacteria;c__Alphaproteobacteria;o__Rhodospirillales;f__unclassified_o__Rhodospirillales;g__unclassified_o__Rhodospirillales	0.0009	0.0128	0.0020	0.0020	0.0019
p__Proteobacteria;c__Betaproteobacteria;o__Burkholderiales;f__unclassified_o__Burkholderiales;g__Piscinibacter	0.0009	0.0015	0.0035	0.0033	0.0029
p__Verrucomicrobia;c__Verrucomicrobiae;o__Verrucomicrobiales;f__Verrucomicrobia_subdivision_3;g__unclassified_f__Verrucomicrobia_subdivision_3	0.0014	0.0013	0.0010	0.0012	0.0011
p__Proteobacteria;c__Betaproteobacteria;o__Burkholderiales;f__unclassified_o__Burkholderiales;g__Rubrivivax	0.0007	0.0012	0.0014	0.0013	0.0012
p__Proteobacteria;c__Betaproteobacteria;o__Burkholderiales;f__Burkholderiaceae;g__Ralstonia	0.0016	0.0007	0.0007	0.0007	0.0005
p__Proteobacteria;c__Betaproteobacteria;o__Burkholderiales;f__Comamonadaceae;g__unclassified_f__Comamonadaceae	0.0013	0.0014	0.0015	0.0013	0.0012
p__Proteobacteria;c__Betaproteobacteria;o__Burkholderiales;f__Comamonadaceae;g__Ramlibacter	0.0015	0.0013	0.0012	0.0011	0.0009
p__Proteobacteria;c__Candidatus_Muproteobacteria;o__unclassified_c__Candidatus_Muproteobacteria;f__unclassified_c__Candidatus_Muproteobacteria;g__unclassified_c__Candidatus_Muproteobacteria	0.0017	0.0097	0.0040	0.0044	0.0034
p__Proteobacteria;c__Betaproteobacteria;o__Burkholderiales;f__unclassified_o__Burkholderiales;g__Ideonella	0.0008	0.0014	0.0015	0.0014	0.0011
p__Firmicutes;c__unclassified_p__Firmicutes;o__unclassified_p__Firmicutes;f__unclassified_p__Firmicutes;g__unclassified_p__Firmicutes	0.0018	0.0013	0.0009	0.0008	0.0008
p__Candidatus_Omnitrophica;c__unclassified_p__Candidatus_Omnitrophica;o__unclassified_p__Candidatus_Omnitrophica;f__unclassified_p__Candidatus_Omnitrophica;g__unclassified_p__Candidatus_Omnitrophica	0.0008	0.0007	0.0004	0.0004	0.0003
p__Armatimonadetes;c__unclassified_p__Armatimonadetes;o__unclassified_p__Armatimonadetes;f__unclassified_p__Armatimonadetes;g__unclassified_p__Armatimonadetes	0.0018	0.0013	0.0009	0.0010	0.0010
p__Proteobacteria;c__Alphaproteobacteria;o__Rhizobiales;f__Xanthobacteraceae;g__unclassified_f__Xanth	0.0008	0.0018	0.0052	0.0060	0.0039

obacteraceae					
p__Proteobacteria;c__Deltaproteobacteria;o__Syntrophobacterales;f__Syntrophaceae;g__Desulfobacca	0.0035	0.0019	0.0014	0.0013	0.0011
p__Proteobacteria;c__Gammaproteobacteria;o__Nevskiales;f__Steroidobacteraceae;g__Steroidobacter	0.0003	0.0006	0.0053	0.0015	0.0087
p__Proteobacteria;c__Hydrogenophilalia;o__Hydrogenophilales;f__unclassified_o__Hydrogenophilales;g__unclassified_o__Hydrogenophilales	0.0034	0.0015	0.0014	0.0013	0.0011
p__Acidobacteria;c__Blastocatellia;o__unclassified_c__Blastocatellia;f__unclassified_c__Blastocatellia;g__unclassified_c__Blastocatellia	0.0018	0.0017	0.0021	0.0021	0.0018
p__Spirochaetes;c__Spirochaetia;o__Spirochaetales;f__unclassified_o__Spirochaetales;g__unclassified_o__Spirochaetales	0.0014	0.0010	0.0002	0.0003	0.0002
p__Proteobacteria;c__Gammaproteobacteria;o__Thiotrichales;f__Thiotrichaceae;g__Thioploca	0.0000	0.0000	0.0000	0.0000	0.0000
p__Proteobacteria;c__Betaproteobacteria;o__Burkholderiales;f__Comamonadaceae;g__Variovorax	0.0011	0.0014	0.0016	0.0015	0.0016
p__Elusimicrobia;c__unclassified_p__Elusimicrobia;o__unclassified_p__Elusimicrobia;f__unclassified_p__Elusimicrobia;g__unclassified_p__Elusimicrobia	0.0008	0.0007	0.0005	0.0006	0.0006
p__Actinobacteria;c__Actinobacteria;o__Corynebacteriales;f__Nocardiaceae;g__Rhodococcus_f__Nocardiaceae	0.0026	0.0010	0.0015	0.0013	0.0009
p__candidate_division_NC10;c__unclassified_p__candidate_division_NC10;o__unclassified_p__candidate_division_NC10;f__unclassified_p__candidate_division_NC10;g__Candidatus_Methylomirabilis	0.0003	0.0003	0.0004	0.0004	0.0003
p__Proteobacteria;c__Betaproteobacteria;o__Rhodocyclales;f__unclassified_o__Rhodocyclales;g__unclassified_o__Rhodocyclales	0.0007	0.0010	0.0010	0.0010	0.0007
p__Proteobacteria;c__Alphaproteobacteria;o__Sphingomonadales;f__Sphingomonadaceae;g__Sphingomonas	0.0003	0.0006	0.0015	0.0009	0.0018
p__Actinobacteria;c__Actinobacteria;o__Propionibacteriales;f__Propionibacteriaceae;g__Brooklawnia	0.0000	0.0001	0.0001	0.0002	0.0002
p__Cyanobacteria;c__unclassified_p__Cyanobacteria;o__Oscillatoriales;f__Microcoleaceae;g__unclassified_f__Microcoleaceae	0.0000	0.0000	0.0006	0.0000	0.0001
p__Acidobacteria;c__Acidobacteriia;o__Bryobacterales;f__unclassified_o__Bryobacterales;g__unclassified_	0.0019	0.0014	0.0011	0.0010	0.0009

o__Bryobacterales					
p__Chrysiogenetes_d__Bacteria;c__Chrysiogenetes_p__Chrysiogenetes;o__Chrysiogenales;f__unclassified_o__Chrysiogenales;g__unclassified_o__Chrysiogenales	0.0022	0.0013	0.0001	0.0001	0.0001
p__Bacteroidetes;c__Bacteroidia;o__Bacteroidales;f__unclassified_o__Bacteroidales;g__unclassified_o__Bacteroidales	0.0017	0.0007	0.0002	0.0005	0.0003
p__Proteobacteria;c__Alphaproteobacteria;o__Rhizobiales;f__Hyphomicrobiaceae;g__Hyphomicrobium	0.0006	0.0010	0.0021	0.0022	0.0019
p__Actinobacteria;c__Rubrobacteria;o__Gaiellales;f__Gaiellaceae;g__Gaiella	0.0008	0.0014	0.0033	0.0038	0.0025
p__Lentisphaerae;c__unclassified_p__Lentisphaerae;o__unclassified_p__Lentisphaerae;f__unclassified_p__Lentisphaerae;g__unclassified_p__Lentisphaerae	0.0012	0.0007	0.0004	0.0004	0.0005
p__Proteobacteria;c__Betaproteobacteria;o__Burkholderiales;f__unclassified_o__Burkholderiales;g__Methylibium	0.0006	0.0010	0.0013	0.0011	0.0010
p__Proteobacteria;c__Betaproteobacteria;o__Burkholderiales;f__Comamonadaceae;g__Curvibacter	0.0059	0.0019	0.0007	0.0005	0.0004
p__Gemmatimonadetes_d__Bacteria;c__Gemmatimonadetes_p__Gemmatimonadetes;o__Gemmatimonadales;f__Gemmatimonadaceae;g__Gemmatimonas	0.0010	0.0011	0.0011	0.0011	0.0009
p__Actinobacteria;c__Actinobacteria;o__Propionibacteriales;f__Nocardioidaceae;g__Nocardioides	0.0007	0.0006	0.0010	0.0009	0.0008
p__Candidatus_Latescibacteria;c__unclassified_p__Candidatus_Latescibacteria;o__unclassified_p__Candidatus_Latescibacteria;f__unclassified_p__Candidatus_Latescibacteria;g__unclassified_p__Candidatus_Latescibacteria	0.0019	0.0011	0.0008	0.0008	0.0007
p__Chloroflexi;c__Thermoflexia;o__unclassified_c__Thermoflexia;f__unclassified_c__Thermoflexia;g__unclassified_c__Thermoflexia	0.0006	0.0005	0.0001	0.0001	0.0001
p__Proteobacteria;c__Gammaproteobacteria;o__Thiotrichales;f__Thiotrichaceae;g__Beggiatoa	0.0000	0.0001	0.0001	0.0000	0.0001
p__Candidatus_Woesebacteria;c__unclassified_p__Candidatus_Woesebacteria;o__unclassified_p__Candidatus_Woesebacteria;f__unclassified_p__Candidatus_Woesebacteria;g__unclassified_p__Candidatus_Woesebacteria	0.0007	0.0020	0.0001	0.0000	0.0000
p__Candidatus_Bipolaricaulota;c__unclassified_p__Candidatus_Bipolaricaulota;o__unclassified_p__Candi	0.0031	0.0007	0.0001	0.0001	0.0001

datus_Bipolaricaulota;f__unclassified_p__Candidatus_Bipolaricaulota;g__unclassified_p__Candidatus_Bipolaricaulota					
other	0.1815	0.1862	0.1877	0.2006	0.2409

Table S3. Relative abundance of bacterial communities at various points along natural river channel C.

#Taxonomy	C1	C2	C3	C4	C5
p__Proteobacteria;c__Deltaproteobacteria;o__unclassified_c__Deltaproteobacteria;f__unclassified_c__Deltaproteobacteria;g__unclassified_c__Deltaproteobacteria	0.1029	0.1144	0.0933	0.0973	0.0985
p__Acidobacteria;c__unclassified_p__Acidobacteria;o__unclassified_p__Acidobacteria;f__unclassified_p__Acidobacteria;g__unclassified_p__Acidobacteria	0.0619	0.0666	0.0682	0.0672	0.0663
p__Proteobacteria;c__Betaproteobacteria;o__unclassified_c__Betaproteobacteria;f__unclassified_c__Betaproteobacteria;g__unclassified_c__Betaproteobacteria	0.1034	0.1086	0.1014	0.0973	0.1010
p__Chloroflexi;c__unclassified_p__Chloroflexi;o__unclassified_p__Chloroflexi;f__unclassified_p__Chloroflexi;g__unclassified_p__Chloroflexi	0.0588	0.0600	0.0565	0.0624	0.0698
p__Proteobacteria;c__Gammaproteobacteria;o__Pseudomonadales;f__Pseudomonadaceae;g__Pseudomonas	0.0237	0.0215	0.0211	0.0223	0.0222
p__Candidatus_Aminicenantes;c__unclassified_p__Candidatus_Aminicenantes;o__unclassified_p__Candidatus_Aminicenantes;f__unclassified_p__Candidatus_Aminicenantes;g__unclassified_p__Candidatus_Aminicenantes	0.0098	0.0099	0.0126	0.0137	0.0109
p__Proteobacteria;c__Gammaproteobacteria;o__unclassified_c__Gammaproteobacteria;f__unclassified_c__Gammaproteobacteria;g__unclassified_c__Gammaproteobacteria	0.0256	0.0266	0.0278	0.0252	0.0312
p__unclassified_d__Bacteria;c__unclassified_d__Bacteria;o__unclassified_d__Bacteria;f__unclassified_d__Bacteria;g__unclassified_d__Bacteria	0.0154	0.0152	0.0158	0.0159	0.0144
p__Planctomycetes;c__unclassified_p__Planctomycetes;o__unclassified_p__Planctomycetes;f__unclassified_p__Planctomycetes;g__unclassified_p__Planctomycetes	0.0123	0.0129	0.0125	0.0118	0.0096

p__Gemmatimonadetes_d__Bacteria;c__unclassified_p__Gemmatimonadetes_d__Bacteria;o__unclassified_p__Gemmatimonadetes_d__Bacteria;f__unclassified_p__Gemmatimonadetes_d__Bacteria;g__unclassified_p__Gemmatimonadetes_d__Bacteria	0.0127	0.0129	0.0128	0.0121	0.0117
p__Spirochaetes;c__unclassified_p__Spirochaetes;o__unclassified_p__Spirochaetes;f__unclassified_p__Spirochaetes;g__unclassified_p__Spirochaetes	0.0132	0.0142	0.0174	0.0160	0.0115
p__Actinobacteria;c__Actinobacteria;o__unclassified_c__Actinobacteria;f__unclassified_c__Actinobacteria;g__unclassified_c__Actinobacteria	0.0168	0.0203	0.0165	0.0180	0.0175
p__Chloroflexi;c__Anaerolineae;o__unclassified_c__Anaerolineae;f__unclassified_c__Anaerolineae;g__unclassified_c__Anaerolineae	0.0087	0.0076	0.0085	0.0098	0.0090
p__Candidatus_Rokubacteria;c__unclassified_p__Candidatus_Rokubacteria;o__unclassified_p__Candidatus_Rokubacteria;f__unclassified_p__Candidatus_Rokubacteria;g__unclassified_p__Candidatus_Rokubacteria	0.0224	0.0261	0.0232	0.0238	0.0208
p__Proteobacteria;c__Alphaproteobacteria;o__Rhizobiales;f__Bradyrhizobiaceae;g__Bradyrhizobium	0.0088	0.0080	0.0090	0.0086	0.0081
p__Proteobacteria;c__Alphaproteobacteria;o__unclassified_c__Alphaproteobacteria;f__unclassified_c__Alphaproteobacteria;g__unclassified_c__Alphaproteobacteria	0.0089	0.0087	0.0082	0.0087	0.0082
p__Actinobacteria;c__unclassified_p__Actinobacteria;o__unclassified_p__Actinobacteria;f__unclassified_p__Actinobacteria;g__unclassified_p__Actinobacteria	0.0076	0.0089	0.0092	0.0101	0.0102
p__Proteobacteria;c__Deltaproteobacteria;o__Myxococcales;f__Anaeromyxobacteraceae;g__Anaeromyxobacter	0.0036	0.0033	0.0036	0.0035	0.0032
p__Nitrospirae;c__unclassified_p__Nitrospirae;o__unclassified_p__Nitrospirae;f__unclassified_p__Nitrospirae;g__unclassified_p__Nitrospirae	0.0072	0.0068	0.0071	0.0076	0.0071
p__Nitrospirae;c__Nitrospira;o__Nitrospirales;f__Nitrospiraceae;g__Nitrospira	0.0114	0.0088	0.0095	0.0094	0.0103
p__Proteobacteria;c__unclassified_p__Proteobacteria;o__unclassified_p__Proteobacteria;f__unclassified_p__Proteobacteria;g__unclassified_p__Proteobacteria	0.0058	0.0059	0.0071	0.0063	0.0074
p__Proteobacteria;c__Gammaproteobacteria;o__Pseudomonadales;f__Moraxellaceae;g__unclassified_f__Moraxellaceae	0.0227	0.0235	0.0187	0.0166	0.0100

p__Proteobacteria;c__Alphaproteobacteria;o__Rhizobiales;f__unclassified_o__Rhizobiales;g__unclassified_o__Rhizobiales	0.0064	0.0072	0.0062	0.0073	0.0068
p__Proteobacteria;c__Betaproteobacteria;o__Rhodocyclales;f__Azonexaceae;g__Dechloromonas	0.0077	0.0088	0.0092	0.0086	0.0058
p__Proteobacteria;c__Deltaproteobacteria;o__Myxococcales;f__unclassified_o__Myxococcales;g__unclassified_o__Myxococcales	0.0058	0.0064	0.0068	0.0061	0.0061
p__Candidatus_Abyssubacteria;c__unclassified_p__Candidatus_Abyssubacteria;o__unclassified_p__Candidatus_Abyssubacteria;f__unclassified_p__Candidatus_Abyssubacteria;g__unclassified_p__Candidatus_Abyssubacteria	0.0002	0.0002	0.0002	0.0002	0.0002
p__Proteobacteria;c__Deltaproteobacteria;o__Desulfobacterales;f__Desulfobacteraceae;g__unclassified_f__Desulfobacteraceae	0.0038	0.0034	0.0054	0.0063	0.0055
p__Proteobacteria;c__Deltaproteobacteria;o__Syntrophobacterales;f__Syntrophaceae;g__unclassified_f__Syntrophaceae	0.0090	0.0093	0.0102	0.0105	0.0074
p__Chloroflexi;c__Anaerolineae;o__Anaerolineales;f__unclassified_o__Anaerolineales;g__unclassified_o__Anaerolineales	0.0033	0.0030	0.0033	0.0037	0.0036
p__Verrucomicrobia;c__unclassified_p__Verrucomicrobia;o__unclassified_p__Verrucomicrobia;f__unclassified_p__Verrucomicrobia;g__unclassified_p__Verrucomicrobia	0.0062	0.0057	0.0068	0.0068	0.0053
p__candidate_division_Zixibacteria;c__unclassified_p__candidate_division_Zixibacteria;o__unclassified_p__candidate_division_Zixibacteria;f__unclassified_p__candidate_division_Zixibacteria;g__unclassified_p__candidate_division_Zixibacteria	0.0008	0.0007	0.0009	0.0009	0.0008
p__Proteobacteria;c__Betaproteobacteria;o__Burkholderiales;f__unclassified_o__Burkholderiales;g__unclassified_o__Burkholderiales	0.0074	0.0076	0.0076	0.0067	0.0064
p__Candidatus_Eisenbacteria;c__unclassified_p__Candidatus_Eisenbacteria;o__unclassified_p__Candidatus_Eisenbacteria;f__unclassified_p__Candidatus_Eisenbacteria;g__unclassified_p__Candidatus_Eisenbacteria	0.0059	0.0061	0.0064	0.0061	0.0052
p__Gemmatimonadetes_d__Bacteria;c__Gemmatimonadetes_p__Gemmatimonadetes;o__Gemmatimonadales;f__unclassified_o__Gemmatimonadales;g__unclassified_o__Gemmatimonadales	0.0045	0.0048	0.0046	0.0043	0.0041

p__Proteobacteria;c__Deltaproteobacteria;o__Desulfuromonadales;f__Desulfuromonadaceae;g__Desulfuromonas	0.0085	0.0078	0.0112	0.0113	0.0076
p__Proteobacteria;c__Deltaproteobacteria;o__Syntrophobacterales;f__unclassified_o__Syntrophobacterales;g__unclassified_o__Syntrophobacterales	0.0074	0.0077	0.0087	0.0095	0.0073
p__Proteobacteria;c__Alphaproteobacteria;o__Rhodospirillales;f__Rhodospirillaceae;g__unclassified_f__Rhodospirillaceae	0.0049	0.0043	0.0043	0.0041	0.0038
p__Chloroflexi;c__Anaerolineae;o__Anaerolineales;f__Anaerolineaceae;g__unclassified_f__Anaerolineaceae	0.0032	0.0029	0.0037	0.0039	0.0036
p__Planctomycetes;c__Phycisphaerae;o__unclassified_c__Phycisphaerae;f__unclassified_c__Phycisphaerae;g__unclassified_c__Phycisphaerae	0.0023	0.0022	0.0023	0.0022	0.0017
p__Chloroflexi;c__Dehalococcoidia;o__unclassified_c__Dehalococcoidia;f__unclassified_c__Dehalococcoidia;g__unclassified_c__Dehalococcoidia	0.0015	0.0015	0.0015	0.0016	0.0018
p__Nitrospirae;c__Nitrospira;o__Nitrospirales;f__Nitrospiraceae;g__unclassified_f__Nitrospiraceae	0.0027	0.0026	0.0027	0.0030	0.0028
p__Proteobacteria;c__Betaproteobacteria;o__Nitrosomonadales;f__unclassified_o__Nitrosomonadales;g__unclassified_o__Nitrosomonadales	0.0027	0.0024	0.0021	0.0019	0.0029
p__candidate_division_NC10;c__unclassified_p__candidate_division_NC10;o__unclassified_p__candidate_division_NC10;f__unclassified_p__candidate_division_NC10;g__unclassified_p__candidate_division_NC10	0.0043	0.0043	0.0049	0.0046	0.0050
p__Acidobacteria;c__Thermoanaerobaculia;o__unclassified_c__Thermoanaerobaculia;f__unclassified_c__Thermoanaerobaculia;g__unclassified_c__Thermoanaerobaculia	0.0020	0.0026	0.0020	0.0020	0.0024
p__Proteobacteria;c__Betaproteobacteria;o__Nitrosomonadales;f__Thiobacillaceae;g__Thiobacillus	0.0016	0.0017	0.0019	0.0018	0.0019
p__Proteobacteria;c__Betaproteobacteria;o__Rhodocyclales;f__Rhodocyclaceae;g__Aromatoleum	0.0048	0.0054	0.0049	0.0048	0.0054
p__Bacteroidetes;c__unclassified_p__Bacteroidetes;o__unclassified_p__Bacteroidetes;f__unclassified_p__Bacteroidetes;g__unclassified_p__Bacteroidetes	0.0026	0.0017	0.0024	0.0027	0.0019
p__Proteobacteria;c__Betaproteobacteria;o__Burkholderiales;f__Burkholderiaceae;g__unclassified_f__Burk	0.0034	0.0035	0.0038	0.0034	0.0031

holderiaceae					
p__Proteobacteria;c__Gammaproteobacteria;o__Xanthomonadales;f__unclassified_o__Xanthomonadales;g__unclassified_o__Xanthomonadales	0.0044	0.0040	0.0043	0.0042	0.0044
p__Proteobacteria;c__Deltaproteobacteria;o__Desulfobacterales;f__unclassified_o__Desulfobacterales;g__unclassified_o__Desulfobacterales	0.0016	0.0015	0.0022	0.0028	0.0022
p__Proteobacteria;c__Deltaproteobacteria;o__Syntrophobacterales;f__Syntrophaceae;g__Syntrophus_f__Syntrophaceae	0.0061	0.0063	0.0057	0.0062	0.0050
p__Planctomycetes;c__Phycisphaerae;o__Phycisphaerales;f__unclassified_o__Phycisphaerales;g__unclassified_o__Phycisphaerales	0.0013	0.0013	0.0013	0.0013	0.0011
p__Proteobacteria;c__Betaproteobacteria;o__Rhodocyclales;f__Rhodocyclaceae;g__unclassified_f__Rhodocyclaceae	0.0048	0.0053	0.0051	0.0049	0.0042
p__Proteobacteria;c__Gammaproteobacteria;o__Xanthomonadales;f__Xanthomonadaceae;g__Stenotrophomonas	0.0019	0.0020	0.0018	0.0018	0.0018
p__Acidobacteria;c__Vicinamibacteria;o__unclassified_c__Vicinamibacteria;f__Vicinamibacteraceae;g__Luteitalea	0.0024	0.0025	0.0023	0.0025	0.0024
p__Planctomycetes;c__Planctomycetia;o__Planctomycetales;f__Planctomycetaceae;g__unclassified_f__Planctomycetaceae	0.0017	0.0017	0.0017	0.0017	0.0014
p__Proteobacteria;c__Deltaproteobacteria;o__Desulfuromonadales;f__Geobacteraceae;g__Geobacter	0.0025	0.0023	0.0027	0.0028	0.0020
p__Proteobacteria;c__Gammaproteobacteria;o__Acidiferrobacterales;f__Acidiferrobacteraceae;g__Sulfuricaulis	0.0004	0.0004	0.0004	0.0005	0.0004
p__Proteobacteria;c__Alphaproteobacteria;o__Rhizobiales;f__Xanthobacteraceae;g__Pseudolabrys	0.0027	0.0027	0.0023	0.0026	0.0026
p__Acidobacteria;c__Holophagae;o__unclassified_c__Holophagae;f__unclassified_c__Holophagae;g__unclassified_c__Holophagae	0.0019	0.0021	0.0025	0.0026	0.0027
p__Acidobacteria;c__Acidobacteriia;o__Bryobacterales;f__Solibacteraceae;g__Candidatus_Solibacter	0.0019	0.0017	0.0018	0.0017	0.0017
p__Proteobacteria;c__Zetaproteobacteria;o__unclassified_c__Zetaproteobacteria;f__unclassified_c__Zetapr	0.0023	0.0023	0.0029	0.0029	0.0024

oteobacteria;g__unclassified_c__Zetaproteobacteria					
p__Proteobacteria;c__Alphaproteobacteria;o__Sphingomonadales;f__Sphingomonadaceae;g__Novosphingobium	0.0055	0.0053	0.0046	0.0044	0.0034
p__Proteobacteria;c__Alphaproteobacteria;o__Rhizobiales;f__Hyphomicrobiaceae;g__Rhodoplanes	0.0013	0.0013	0.0012	0.0013	0.0011
p__Proteobacteria;c__Alphaproteobacteria;o__Rhodospirillales;f__unclassified_o__Rhodospirillales;g__unclassified_o__Rhodospirillales	0.0016	0.0016	0.0014	0.0015	0.0013
p__Proteobacteria;c__Betaproteobacteria;o__Burkholderiales;f__unclassified_o__Burkholderiales;g__Piscinibacter	0.0032	0.0028	0.0032	0.0027	0.0026
p__Verrucomicrobia;c__Verrucomicrobiae;o__Verrucomicrobiales;f__Verrucomicrobia_subdivision_3;g__unclassified_f__Verrucomicrobia_subdivision_3	0.0024	0.0021	0.0029	0.0027	0.0022
p__Proteobacteria;c__Betaproteobacteria;o__Burkholderiales;f__unclassified_o__Burkholderiales;g__Rubrivivax	0.0043	0.0044	0.0043	0.0036	0.0033
p__Proteobacteria;c__Betaproteobacteria;o__Burkholderiales;f__Burkholderiaceae;g__Ralstonia	0.0015	0.0018	0.0012	0.0015	0.0012
p__Proteobacteria;c__Betaproteobacteria;o__Burkholderiales;f__Comamonadaceae;g__unclassified_f__Comamonadaceae	0.0043	0.0039	0.0038	0.0033	0.0030
p__Proteobacteria;c__Betaproteobacteria;o__Burkholderiales;f__Comamonadaceae;g__Ramlibacter	0.0047	0.0042	0.0037	0.0032	0.0030
p__Proteobacteria;c__Candidatus_Muproteobacteria;o__unclassified_c__Candidatus_Muproteobacteria;f__unclassified_c__Candidatus_Muproteobacteria;g__unclassified_c__Candidatus_Muproteobacteria	0.0004	0.0004	0.0004	0.0004	0.0005
p__Proteobacteria;c__Betaproteobacteria;o__Burkholderiales;f__unclassified_o__Burkholderiales;g__Ideonella	0.0039	0.0038	0.0038	0.0033	0.0031
p__Firmicutes;c__unclassified_p__Firmicutes;o__unclassified_p__Firmicutes;f__unclassified_p__Firmicutes;g__unclassified_p__Firmicutes	0.0012	0.0012	0.0014	0.0012	0.0012
p__Candidatus_Omnitrophica;c__unclassified_p__Candidatus_Omnitrophica;o__unclassified_p__Candidatus_Omnitrophica;f__unclassified_p__Candidatus_Omnitrophica;g__unclassified_p__Candidatus_Omnitrophica	0.0011	0.0009	0.0010	0.0011	0.0007

p__Armatimonadetes;c__unclassified_p__Armatimonadetes;o__unclassified_p__Armatimonadetes;f__unclassified_p__Armatimonadetes;g__unclassified_p__Armatimonadetes	0.0014	0.0013	0.0013	0.0013	0.0013
p__Proteobacteria;c__Alphaproteobacteria;o__Rhizobiales;f__Xanthobacteraceae;g__unclassified_f__Xanthobacteraceae	0.0010	0.0010	0.0008	0.0010	0.0008
p__Proteobacteria;c__Deltaproteobacteria;o__Syntrophobacterales;f__Syntrophaceae;g__Desulfobacca	0.0005	0.0006	0.0009	0.0014	0.0012
p__Proteobacteria;c__Gammaproteobacteria;o__Nevskiales;f__Steroidobacteraceae;g__Steroidobacter	0.0011	0.0010	0.0013	0.0009	0.0016
p__Proteobacteria;c__Hydrogenophilalia;o__Hydrogenophilales;f__unclassified_o__Hydrogenophilales;g__unclassified_o__Hydrogenophilales	0.0011	0.0011	0.0011	0.0011	0.0011
p__Acidobacteria;c__Blastocatellia;o__unclassified_c__Blastocatellia;f__unclassified_c__Blastocatellia;g__unclassified_c__Blastocatellia	0.0013	0.0012	0.0013	0.0013	0.0013
p__Spirochaetes;c__Spirochaetia;o__Spirochaetales;f__unclassified_o__Spirochaetales;g__unclassified_o__Spirochaetales	0.0012	0.0012	0.0016	0.0015	0.0010
p__Proteobacteria;c__Gammaproteobacteria;o__Thiotrichales;f__Thiotrichaceae;g__Thioploca	0.0016	0.0013	0.0038	0.0035	0.0107
p__Proteobacteria;c__Betaproteobacteria;o__Burkholderiales;f__Comamonadaceae;g__Variovorax	0.0026	0.0024	0.0025	0.0021	0.0020
p__Elusimicrobia;c__unclassified_p__Elusimicrobia;o__unclassified_p__Elusimicrobia;f__unclassified_p__Elusimicrobia;g__unclassified_p__Elusimicrobia	0.0017	0.0015	0.0015	0.0016	0.0010
p__Actinobacteria;c__Actinobacteria;o__Corynebacteriales;f__Nocardiaceae;g__Rhodococcus_f__Nocardiaceae	0.0006	0.0006	0.0005	0.0008	0.0007
p__candidate_division_NC10;c__unclassified_p__candidate_division_NC10;o__unclassified_p__candidate_division_NC10;f__unclassified_p__candidate_division_NC10;g__Candidatus_Methylomirabilis	0.0013	0.0009	0.0015	0.0011	0.0120
p__Proteobacteria;c__Betaproteobacteria;o__Rhodocyclales;f__unclassified_o__Rhodocyclales;g__unclassified_o__Rhodocyclales	0.0026	0.0028	0.0027	0.0026	0.0024
p__Proteobacteria;c__Alphaproteobacteria;o__Sphingomonadales;f__Sphingomonadaceae;g__Sphingomonas	0.0039	0.0026	0.0022	0.0021	0.0022
p__Actinobacteria;c__Actinobacteria;o__Propionibacteriales;f__Propionibacteriaceae;g__Brooklawnia	0.0000	0.0001	0.0001	0.0001	0.0001

p__Cyanobacteria;c__unclassified_p__Cyanobacteria;o__Oscillatoriales;f__Microcoleaceae;g__unclassified_f__Microcoleaceae	0.0000	0.0000	0.0000	0.0000	0.0001
p__Acidobacteria;c__Acidobacteriia;o__Bryobacterales;f__unclassified_o__Bryobacterales;g__unclassified_o__Bryobacterales	0.0017	0.0015	0.0018	0.0017	0.0016
p__Chrysiogenetes_d__Bacteria;c__Chrysiogenetes_p__Chrysiogenetes;o__Chrysiogenales;f__unclassified_o__Chrysiogenales;g__unclassified_o__Chrysiogenales	0.0009	0.0007	0.0012	0.0013	0.0012
p__Bacteroidetes;c__Bacteroidia;o__Bacteroidales;f__unclassified_o__Bacteroidales;g__unclassified_o__Bacteroidales	0.0008	0.0004	0.0008	0.0009	0.0006
p__Proteobacteria;c__Alphaproteobacteria;o__Rhizobiales;f__Hyphomicrobiaceae;g__Hyphomicrobium	0.0014	0.0016	0.0012	0.0016	0.0014
p__Actinobacteria;c__Rubrobacteria;o__Gaiellales;f__Gaiellaceae;g__Gaiella	0.0009	0.0009	0.0007	0.0008	0.0010
p__Lentisphaerae;c__unclassified_p__Lentisphaerae;o__unclassified_p__Lentisphaerae;f__unclassified_p__Lentisphaerae;g__unclassified_p__Lentisphaerae	0.0011	0.0010	0.0012	0.0011	0.0009
p__Proteobacteria;c__Betaproteobacteria;o__Burkholderiales;f__unclassified_o__Burkholderiales;g__Methylibium	0.0024	0.0024	0.0023	0.0022	0.0019
p__Proteobacteria;c__Betaproteobacteria;o__Burkholderiales;f__Comamonadaceae;g__Curvibacter	0.0010	0.0010	0.0010	0.0010	0.0009
p__Gemmatimonadetes_d__Bacteria;c__Gemmatimonadetes_p__Gemmatimonadetes;o__Gemmatimonadales;f__Gemmatimonadaceae;g__Gemmatimonas	0.0010	0.0010	0.0009	0.0008	0.0008
p__Actinobacteria;c__Actinobacteria;o__Propionibacteriales;f__Nocardioidaceae;g__Nocardioides	0.0061	0.0011	0.0009	0.0010	0.0011
p__Candidatus_Latescibacteria;c__unclassified_p__Candidatus_Latescibacteria;o__unclassified_p__Candidatus_Latescibacteria;f__unclassified_p__Candidatus_Latescibacteria;g__unclassified_p__Candidatus_Latescibacteria	0.0006	0.0008	0.0008	0.0008	0.0007
p__Chloroflexi;c__Thermoflexia;o__unclassified_c__Thermoflexia;f__unclassified_c__Thermoflexia;g__unclassified_c__Thermoflexia	0.0001	0.0001	0.0001	0.0002	0.0001
p__Proteobacteria;c__Gammaproteobacteria;o__Thiotrichales;f__Thiotrichaceae;g__Beggiatoa	0.0012	0.0009	0.0027	0.0026	0.0078
p__Candidatus_Woesebacteria;c__unclassified_p__Candidatus_Woesebacteria;o__unclassified_p__Candid	0.0003	0.0002	0.0002	0.0002	0.0001

atus_Woesebacteria;f__unclassified_p__Candidatus_Woesebacteria;g__unclassified_p__Candidatus_Woesebacteria					
p__Candidatus_Bipolaricaulota;c__unclassified_p__Candidatus_Bipolaricaulota;o__unclassified_p__Candidatus_Bipolaricaulota;f__unclassified_p__Candidatus_Bipolaricaulota;g__unclassified_p__Candidatus_Bipolaricaulota	0.0004	0.0006	0.0007	0.0007	0.0006
other	0.2105	0.1917	0.2070	0.2039	0.2116

Table S4. Relative abundance of secondary functions at various points along natural river channel A.

Secondary function	A1	A2	A3	A4	A5	Primary function
global and overview maps	27.46%	27.85%	27.57%	27.36%	27.52%	L1
carbohydrate metabolism	9.76%	9.71%	9.43%	9.18%	9.28%	L1
amino acid metabolism	7.83%	7.97%	8.04%	8.30%	8.05%	L1
energy metabolism	7.44%	7.49%	7.52%	7.58%	7.53%	L1
metabolism of cofactors and vitamins	4.68%	4.83%	4.81%	4.82%	4.85%	L1
membrane transport	4.31%	4.11%	4.16%	4.26%	4.15%	L3
cellular community – prokaryotes	3.89%	3.87%	3.88%	3.98%	3.86%	L4
signal transduction	3.28%	3.28%	3.32%	3.51%	3.51%	L3
translation	3.53%	3.60%	3.69%	3.50%	3.46%	L2
nucleotide metabolism	2.89%	2.86%	2.98%	2.90%	2.92%	L1
replication and repair	2.64%	2.58%	2.67%	2.58%	2.60%	L2
lipid metabolism	2.31%	2.31%	2.28%	2.27%	2.26%	L1
metabolism of other amino acids	2.08%	2.09%	2.05%	2.09%	2.06%	L1
folding, sorting, and degradation	1.99%	1.96%	2.02%	2.05%	2.05%	L2
xenobiotics biodegradation and metabolism	1.56%	1.48%	1.52%	1.68%	1.59%	L1
glycan biosynthesis and metabolism	1.96%	1.93%	1.85%	1.60%	1.82%	L1
biosynthesis of other secondary metabolites	1.61%	1.71%	1.64%	1.58%	1.63%	L1

Table S5. Relative abundance of secondary functions at various points along natural river channel B.

Secondary function	B1	B2	B3	B4	B5	Primary function
global and overview maps	27.27%	27.18%	27.18%	27.23%	26.98%	L1
carbohydrate metabolism	9.32%	8.93%	8.90%	8.95%	8.84%	L1
amino acid metabolism	8.12%	8.38%	8.82%	8.70%	8.70%	L1
energy metabolism	7.48%	7.35%	7.30%	7.37%	6.98%	L1
metabolism of cofactors and vitamins	4.60%	4.73%	4.57%	4.56%	4.55%	L1
membrane transport	4.44%	4.39%	4.33%	4.27%	4.39%	L3
cellular community – prokaryotes	3.97%	4.00%	3.91%	3.82%	3.91%	L4
signal transduction	3.57%	3.67%	3.69%	3.66%	3.94%	L3
translation	3.11%	2.98%	2.81%	2.83%	2.69%	L2
nucleotide metabolism	2.91%	2.84%	2.81%	2.79%	2.76%	L1
replication and repair	2.50%	2.46%	2.28%	2.30%	2.27%	L2
lipid metabolism	2.41%	2.39%	2.45%	2.49%	2.49%	L1
metabolism of other amino acids	2.23%	2.32%	2.50%	2.47%	2.52%	L1
folding, sorting, and degradation	1.98%	1.98%	1.93%	1.97%	1.86%	L2
xenobiotics biodegradation and metabolism	1.75%	1.93%	2.20%	2.17%	2.26%	L1
glycan biosynthesis and metabolism	1.79%	1.68%	1.53%	1.58%	1.55%	L1
biosynthesis of other secondary metabolites	1.57%	1.52%	1.58%	1.56%	1.58%	L1

Table S6. Relative abundance of secondary functions at various points along natural river channel C.

Secondary function	C1	C2	C3	C4	C5	Primary function
global and overview maps	26.90%	26.95%	26.85%	26.97%	26.95%	L1
carbohydrate metabolism	9.10%	8.99%	9.10%	9.14%	9.06%	L1
amino acid metabolism	8.70%	8.75%	8.63%	8.65%	8.57%	L1

Secondary function	C1	C2	C3	C4	C5	Primary function
energy metabolism	7.88%	7.79%	7.84%	7.87%	7.89%	L1
metabolism of cofactors and vitamins	4.40%	4.47%	4.36%	4.43%	4.53%	L1
membrane transport	4.31%	4.37%	4.31%	4.36%	4.35%	L3
cellular community – prokaryotes	3.89%	3.93%	3.90%	3.95%	3.92%	L4
signal transduction	3.44%	3.40%	3.45%	3.37%	3.40%	L3
translation	3.23%	3.22%	3.24%	3.22%	3.21%	L2
nucleotide metabolism	2.93%	2.91%	2.93%	2.91%	2.90%	L1
replication and repair	2.43%	2.43%	2.44%	2.42%	2.50%	L2
lipid metabolism	2.38%	2.41%	2.35%	2.34%	2.34%	L1
metabolism of other amino acids	2.25%	2.26%	2.29%	2.25%	2.23%	L1
folding, sorting, and degradation	2.18%	2.12%	2.15%	2.17%	2.19%	L2
xenobiotics biodegradation and metabolism	2.00%	2.01%	2.00%	1.97%	1.91%	L1
glycan biosynthesis and metabolism	1.36%	1.37%	1.41%	1.38%	1.42%	L1
biosynthesis of other secondary metabolites	1.40%	1.43%	1.41%	1.45%	1.40%	L1
